# A systematic scoping review of mentoring support on professional identity formation

**DOI:** 10.1186/s12909-024-06357-3

**Published:** 2024-11-27

**Authors:** Lalit Kumar Radha Krishna, Hannah Yi Fang Kwok, Nila Ravindran, Xuan Yu Tan, Jasper Soh, Darius Wei Jun Wan, Varsha Rajalingam, Jun Kiat Lua, Elizabeth Yong Mei Leong, Tiat Yan Low, Aiden Wei-Jun Chan, Chong Jin Nicholas Lim, Yen Kit Ng, Arthena Anushka Thenpandiyan, Adele Yi Dawn Lim, Leia Ning Tse, Sriram PL, Sri Priyanka Rajanala, Jun Rey Leong, Elaine Li Ying Quah, Victoria Jia En Fam, Ranitha Govindasamy, Nur Amira Binte Abdul Hamid, Crystal Lim, Dorsett Shin Wei Sim, Eng Koon Ong, Stephen Mason, Nagavalli Somasundaram, Simon Yew Kuang Ong

**Affiliations:** 1 Yong Loo Lin School of Medicine, National University of Singapore, NUHS Tower Block, Level 11, Block 1E, Kent Ridge Road, Singapore, 119228 Singapore; 2 Division of Supportive and Palliative Care, National Cancer Centre Singapore, 30 Hospital Boulevard, Singapore, 168583 Singapore; 3 Division of Cancer Education, National Cancer Centre Singapore, 30 Hospital Boulevard, Singapore, 168583 Singapore; 4 Duke-NUS Medical School, National University of Singapore, 8 College Road, Singapore, 169857 Singapore; 5 Centre for Biomedical Ethics, National University of Singapore, Block MD11, 10 Medical Drive, #02-03, Singapore, 117597 Singapore; 6 Palliative Care Institute Liverpool, Academic Palliative & End of Life Care Centre, Cancer Research Centre, University of Liverpool, 200 London Road, Liverpool, L3 9TA UK; 7 Health Data Science, University of Liverpool, Whelan Building, The Quadrangle, Brownlow Hill, Liverpool, L69 3GB UK; 8 The Palliative Care Centre for Excellence in Research and Education, PalC, Dover Park Hospice, 10 Jalan Tan Tock Seng, Singapore, 308436 Singapore; 9 Lee Kong Chian School of Medicine, Nanyang Technological University, 11 Mandalay Road, Singapore, 308207 Singapore; 10 Division of Psychosocial Oncology, National Cancer Centre Singapore, 30 Hospital Boulevard, Singapore, 168583 Singapore; 11 Medical Social Services, Singapore General Hospital, Outram Road, Singapore, 169608 Singapore; 12 Geylang Polyclinic, National Healthcare Group Polyclinics, 21 Geylang East Central, Singapore, 389707 Singapore; 13 Assisi Hospice, 832 Thomson Road, Singapore, 574627 Singapore; 14 Division of Medical Oncology, National Cancer Centre Singapore, 30 Hospital Boulevard, Singapore, 168583 Singapore

**Keywords:** Mentoring, Mentoring relationships, Professional identity formation, Supervision, Coaching, Role modelling, Mentor, Mentoring support, Apprenticeship, Medical schools, Medicine

## Abstract

**Background:**

Mentoring’s success in nurturing professional identity formation (PIF) has been attributed to its ability to build personalised and enduring mentoring relationships. However, beyond functioning as communities of practice (CoPs) supporting socialisation processes, how mentoring integrates programme values and instils a shared identity amongst mentees remains unclear. The need for personalised guidance and timely attention to a mentee’s unique needs in evolving mentoring relationships point to the critical role of support mechanisms (‘mentoring support’). We conducted a systematic scoping review (SSR) studying “What is known about mentoring support’s role in nurturing PIF?”.

**Methods:**

Adopting PRISMA-ScR guidelines, this SSR was guided by the Systematic Evidence-Based Approach (SEBA). Independent searches were carried out on publications featured between 1st January 2000 and 30th June 2023 in PubMed, Embase, ERIC and Scopus databases. The Split Approach saw concurrent, independent thematic and content analyses of the included articles. The Jigsaw Perspective combined complementary themes and categories, creating broader themes/categories. The subsequent Funnelling Process formed key domains that platformed the synthesis of the discussion.

**Results:**

Two thousand three hundred forty-one abstracts were reviewed, 323 full-text articles were appraised and 151 articles were included and analysed. The key domains identified were (1) definitions and roles; (2) personalisation; (3) shepherding; and (4) PIF.

**Conclusion:**

The success of mentoring in PIF lies in its ability to blend role modelling, supervision, mentoring, coaching and teaching, with self-care, guided reflection, apprenticeship and assessment to meet the individual needs of the mentee and their changing circumstances. Blending the contents of the mentoring umbrella emphasises the critical role of the mentor and host organisation in supporting mentor training, communications, support and assessment mechanisms. Mentee engagement and its active role in support measures complement the CoP-like mentoring programme’s use of blending mentoring support to advance the socialisation process. These insights reflect a complex interactive process scaffolding the development of mentoring relationships and PIF. The effect of the mentoring umbrella on clinical practice requires further study.

**Supplementary Information:**

The online version contains supplementary material available at 10.1186/s12909-024-06357-3.

## Background

Mentoring plays an expanding role in medical education by virtue of its ability to personalise learning experiences and adapt to a mentee’s wide-ranging needs [1–8]. Krishna et al. [9] suggest that much of mentoring’s success is attributed to its relationships and their capacity to build enduring and personalised mentoring interactions that steer how medical students and physicians think, feel and act like professionals, or their professional identity formation (PIF). The two are increasingly seen as interlinked.

Supporting mentoring relationships’ ability to shape PIF is the mentoring programme’s ability to function like a community of practice (CoP) and support the socialisation process. Meeting these two Cruess-ian requirements for the nurturing of PIF has hailed the role of mentoring in fostering PIF. Here, Cruess-ian requisites relate to Cruess et al.’s [10] posit that PIF requires a CoP to scaffold the socialisation process, described as the internalisation of programme belief systems.

However, more recent studies have suggested that there is more to mentoring than functioning as a CoP, or a “persistent, sustaining social network of individuals who share and develop an overlapping knowledge base, set of beliefs, values and history and experiences focused on a common practice and/or enterprise” [11]. Questions about the mentoring relationship’s operationalisation of supporting the socialisation process, the second Cruess-ian requirement, are increasingly being asked. This is due to greater recognition that the instillation of programme beliefs, norms, principles and expectations (belief systems) along the mentee’s journey that moves the mentee from layperson to skilled professional can be met when this process is so highly individualised.

A clue to this missing link lies with Cruess et al.'s [10] acknowledgement that as a personalised process affected by “who they are” at the beginning and “who they wish to become,” PIF requires an individualised approach [12]. The mentoring relationship provides just such a personalised approach through use of the mentoring umbrella [6, 9]. The mentoring umbrella blends role modelling, teaching, tutoring and guided immersion; shepherds meaning-making and psycho-emotional states; fosters coping strategies; supports maturing relationship; supervises developing competencies; guides reflections; and coaches desired competencies and timely remediation [6, 9]. This personalised blend is called Personalised, Appropriate and Longitudinal mentoring support (PAL mentoring support) [6, 9].

Toh et al. [13] suggest that the PAL mentoring support system [14, 15] integrates the programme’s shared values, principles and identity (belief systems) into their self-concepts of personhood, or ‘what makes you, you,’—changing the mentee’s self-identity [8, 16, 17]. However, there is little known about PAL mentoring support [18–23]. To address this gap and guide the design of education programmes beyond mentoring programmes, a systematic scoping review (SSR) on “What is known about mentoring support’s role in nurturing PIF?” was conducted.

## Methods

### Theoretical lens

We employed the Systematic Evidence-Based Approach (SEBA) to guide our SSR, given its established role in evaluating mentoring practice in medical education [24, 25] and its compliance with the PRISMA-ScR guidelines. The SSR in SEBA is guided by a constructivist ontological lens, a paradigm that explores how our perceptions of realities or identities are constructed and understood. This encapsulates the psychosocial, cultural and historical influences that underpin the creation of individual concepts of PIF. Further, the SEBA methodology is supplemented by a relativist epistemological perspective, an approach to understanding knowledge that suggests our viewpoints and beliefs are shaped by unique cultural, social or personal contexts, rather than by any universal, objective truth. Our use of the SEBA methodology thus considers multiple perspectives from quantitative and qualitative data and knowledge synthesis articles in this review [26, 27].

The SEBA methodology’s constructivist ontological and relativist epistemological lens also serves another critical role. It supports the idea that a developing sense of identity is the product of the inculcation of new belief systems into self-concepts of personhood. This process can be visualised through the use of the Ring Theory of Personhood (RToP) that captures the integration of new belief systems into the mentee’s current belief systems contained within the four aspects of their sense of personhood. The effects of these changes in belief systems in one or more aspects of the mentee’s sense of personhood lead to changes in their identity. These changes are captured by the Krishna-Pisupati Model for Professional Identity Formation (henceforth KPM).

## The ring theory of personhood

The mentoring relationship’s ability to shape PIF lies in the intimate relationship between belief systems, self-concepts of personhood and notions of self-identity. The RToP captures changes in an individual’s belief systems during the socialisation process. Progressing through the structured mentoring trajectory across competency-based mentoring stages and within the curated mentoring environment of the CoP-like mentoring programme introduces the mentee to new belief systems. Guided immersion into the practice and along the mentoring trajectory; guided reflections; mentored meaning-making; timely and personalised feedback; and supervised debriefs enable the integration of these new experiences, insights and reflections into the mentee’s current belief systems.

**Fig. 1:**
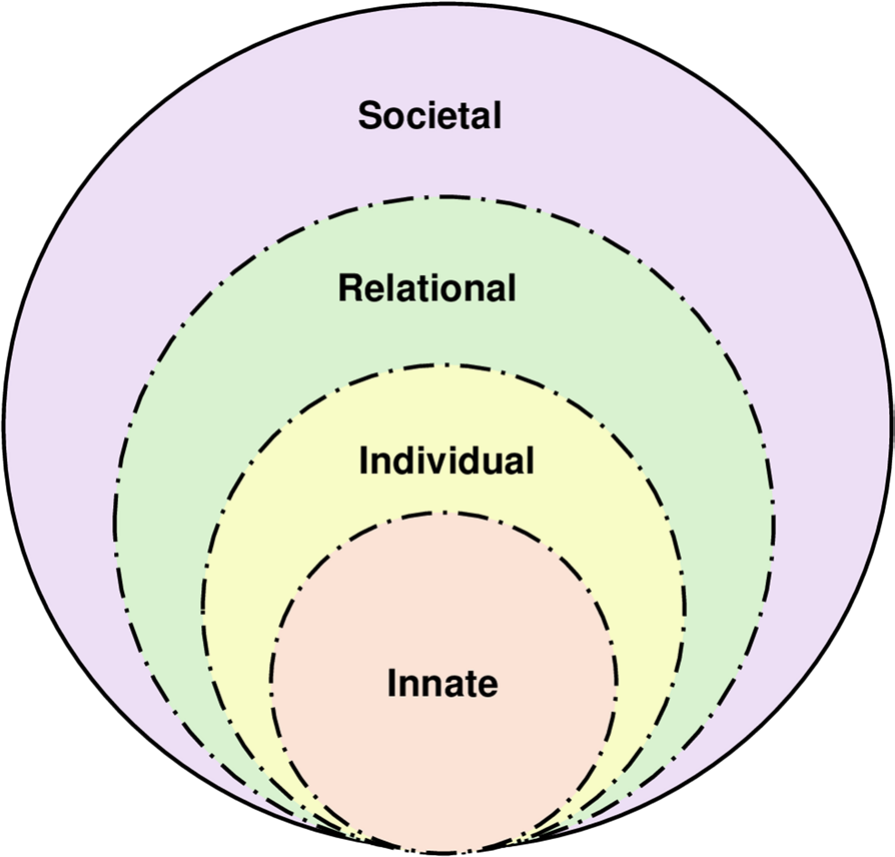
The Ring Theory of Personhood

The RToP [2, 13, 28] suggests that there four aspects of personhood—the Innate, Individual, Relational and Societal aspects of personhood (Fig. 1). Each aspect is accompanied by a corresponding belief system [28].

Changes in the belief systems surrounding existentiality and spirituality in the Innate Ring; emotions, thoughts and thinking in the Individual Ring; the nurturing of close personal ties in the Relational Ring; and the maintenance of social expectations, cultural norms and professional standards in the Societal Ring, shape the mentee’s self-concepts of personhood [28]. In turn, modifications to the individual’s sense of self are quintessential in ensuring congruence, which is the unity between current belief systems, self-perceptions of personhood and identity, and social validation which involves current practice settings, programme culture and academic structure.

**Fig. 2:**
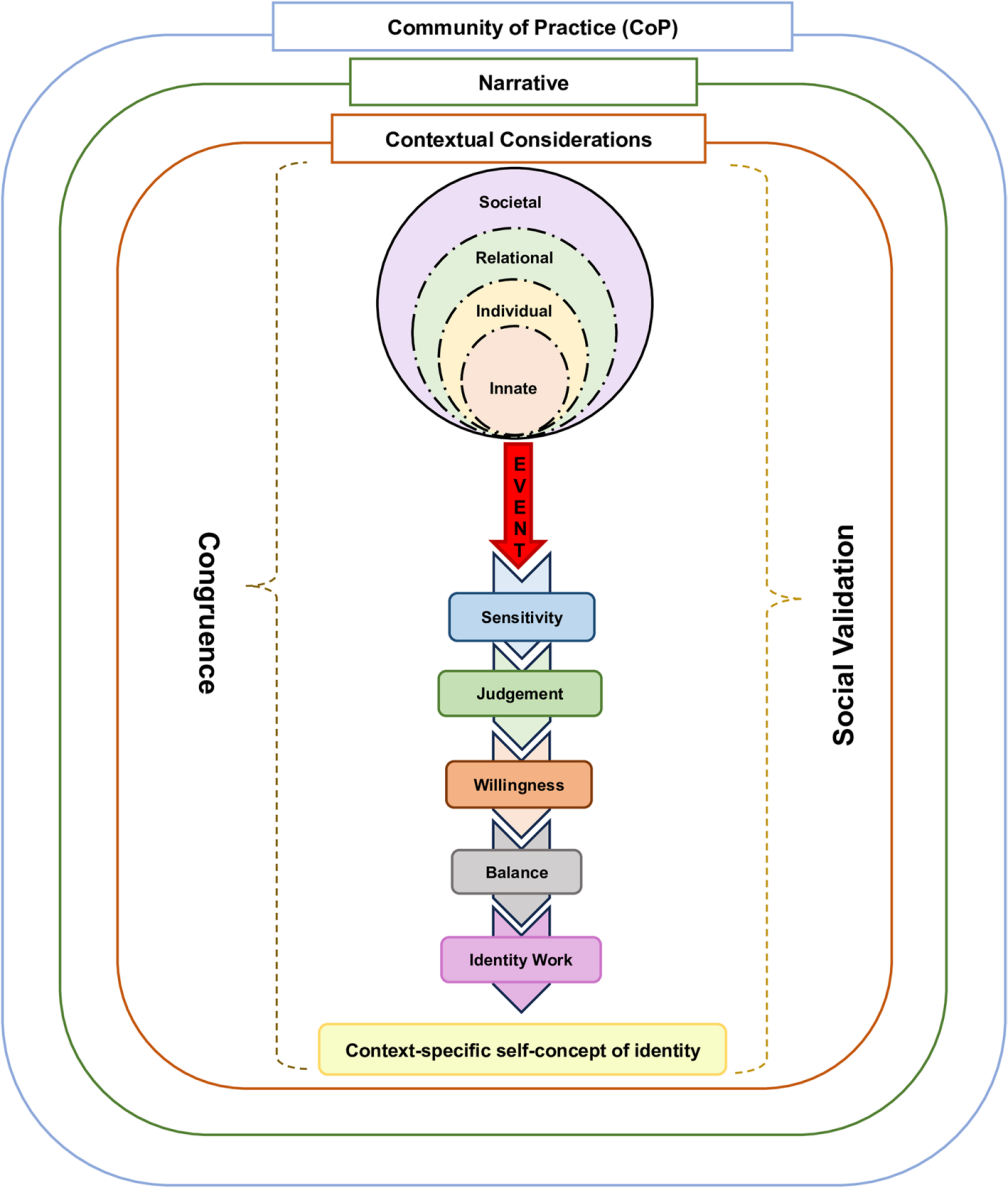
The Krishna-Pisupati Model for Professional Identity Formation

The KPM (Fig. 2) captures the nuances of the complex ‘balancing’ process behind what and how these new belief systems are integrated. Awareness of ‘sensitivity’ to these changes or an ‘event’ triggers a determination as to the significance of the ‘event’ (‘judgement’), as well as an internal evaluation of the ‘willingness’ to address the ‘event’ and the individual’s ability, experience, and opportunity to do so [3]. ‘Balancing’ these sometimes-competing considerations behind the development of a context-specific self-concept of identity [2, 3, 16] is impacted by access to PAL mentoring support [3, 16]. The shifts in context-specific identity reflect the developing professional identity [28].

### Stage 1 of SEBA: systematic approach

**Fig. 3:**
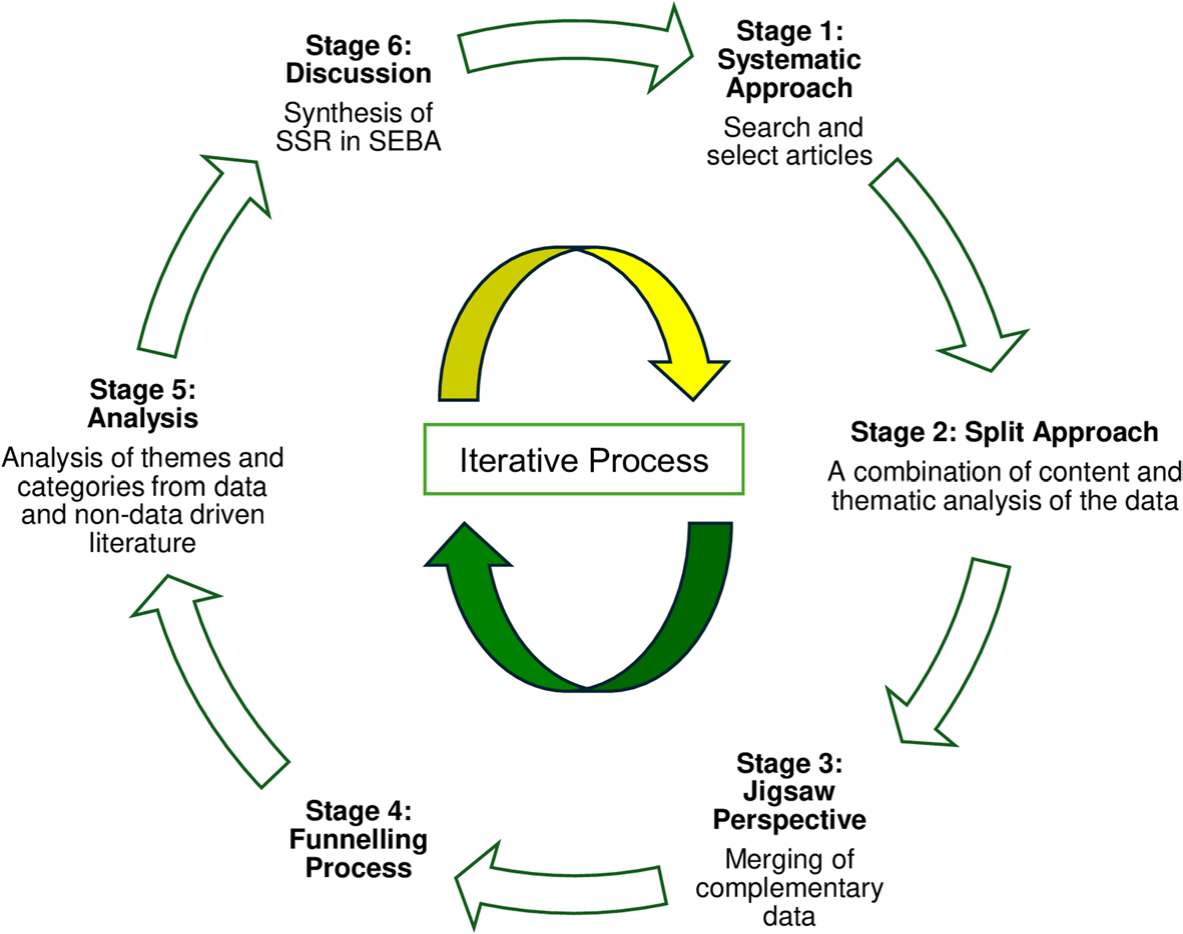
The Systematic Evidenced-Based Approach (SEBA)

The stages of SEBA are outlined in Fig. 3.

The Population, Intervention, Comparison, Outcome and Study Design (PICOS) framework [29] was used to navigate the primary research question, “What is known about mentoring support?” and secondary question, “What is known of the features scaffolding the use of mentoring support and its impact on PIF?” (Table 1).

**Table 1:** Population, Intervention, Comparison, Outcome and Study Design (PICOS) Framework, Inclusion and Exclusion Criteria Applied to Database Search

PICOs	Inclusion	Exclusion
**Population**	Junior physicians, residents and medical students	Allied health specialties (e.g. nursing, physiotherapy, occupational therapy)
**Intervention**	Accounts of mentoring support involving junior physicians, residents and/or medical students mentored by seniors aimed at advancing professional and/or personal development of the mentee • Mentoring processes • Mentor factors • Mentoring relationship • Role of stakeholders and organizations • Outcomes of mentoring • Barriers to mentoring	Peer mentoring, mentoring patients, or mentoring by patients.
**Comparison**	Comparisons between mentoring programmes, editorials, perspectives, reflective, narratives and opinion pieces	
**Outcome**	Personal, professional, research and academic outcomes and impact on PIF	
**Study design**	All study designs are included	Systematic review, literature reviews and narrative reviews

In view of time and manpower limitations, the research team conducted searches independently between 18th October 2023 and 17th January 2024 on PubMed, Embase, ERIC and Scopus databases for articles published between 1st January 2000 and 31st December 2023 (refer to Additional File 1 for the full database search strategy). Upon agreement on the shortlisted articles, the research team subsequently reviewed and summarised the articles to capture pertinent details of mentoring support mechanisms [30].

**Fig. 4:**
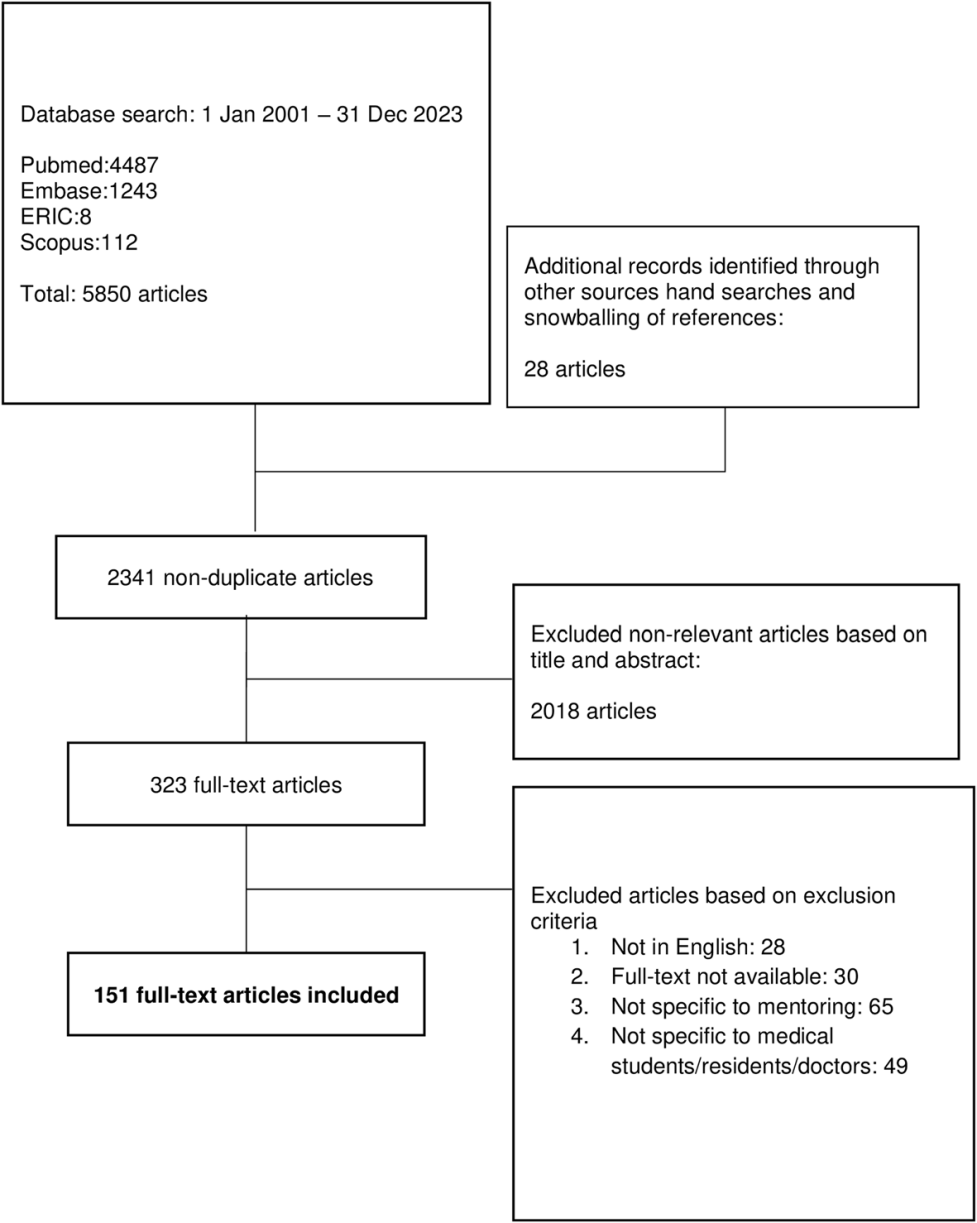
PRISMA Flowchart

A total of 2341 abstracts were reviewed, 323 full-text articles were appraised and 151 articles were included and analysed (Fig. 4).

### Stage 2 of SEBA: split approach

Data analysis of the shortlisted articles was divided between two independent teams of researchers. The first team performed Braun and Clarke’s [31] approach to thematic analysis to identify key themes in the data [32]. Simultaneously, the second team employed Hsieh and Shannon’s [31] method to directed content analysis to determine and operationalise a priori coding categories from mentoring studies by Krishna et al. [6] and Toh et al. [13]. This dual approach accounted for the limitations of each method of data analysis, including the subjectivity in thematic analysis and the lack of depth in directed content analysis [2, 33]. Resultantly, a more holistic and refined analysis of the data was captured.

### Stage 3 of SEBA: jigsaw perspective

Like pieces of a jigsaw puzzle, overlapping or complementary findings from both methods were combined to create bigger puzzle pieces, called themes/categories [34, 35].

### Stage 4 of SEBA: funnelling process

The identified themes/categories were then compared with the aforementioned article summaries to ensure that the research teams effectively retained pivotal information and curtailed omissions [13]. This process directed the funnelling of key domains that formed the basis of the ensuing discussion.

### Stage 5 of SEBA: analysis of evidence-based and non-data-driven literature

To ameliorate concerns about the non-data-based literature biasing the study, the research team compared the themes from evidenced-based publications with those from non-data-based articles [13]. Similar themes found in both groups suggest the analysis was not biased by non-data-based literature.

## Results

The themes identified were (1) constituents, and (2) approaches. The categories were (1) PIF; (2) approach; (3) role; and (4) shepherding.

The domains created by combining the subthemes and subcategories identified were (1) definitions and roles; (2) personalisation; (3) shepherding; and (4) professional identity formation. These domains draw a relationship between mentoring and PIF.

### Domain 1. Definitions and roles

Current definitions of the various elements ascribed to mentoring support are summarised in Table 2.

**Table 2:** Definitions/Descriptions of mentoring support elements

Elements of Mentoring Support	Definitions
**Mentoring**	▪ “Dynamic, context-dependent, goal-sensitive, mutually-beneficial relationship between an experienced clinician and junior clinicians and/or undergraduates that is focused upon advancing the development of the mentee” [36].
**Teaching**	▪ Impart knowledge and guide studies by precept, examples or experience [36]. ▪ Teaching in the clinical environment is defined as teaching and learning focused on and usually directly involving patients and their problems [37].
**Role Modelling**	▪ Active observation of the role model’s personal, clinical and/or social circumstances; practice, attitudes, decisions and skills; reflection on these observations; translation of these insights into principles and actions; and integration of these insights into practice, thinking, attitudes, skills, deliberations and conduct [26].
**Coaching**	▪ An inherently creative activity of bringing forth knowledge, wisdom and insight [38]. ▪ A coach works with a student to continually improve his/her performance, usually on areas that the student deems weak [39]. ▪ Involves asking questions [39]; listening deeply [38]; keenly observing [38, 40]; evaluating and identifying gaps [41]; providing specific and concrete feedback [40, 41]; creating goals; exploring solutions and holding the individual accountable [41]; supporting reflection [38, 42]; setting goals [42]; developing a comprehensive study plan [42]; and ensuring a commitment to learning [38]. ▪ May improve emotional intelligence, durability, wellbeing and resilience [39] . ▪ In medical education, two main types of coaching have been described [41]: (a) Coaching in clinical skills: coach directly observes the learner in the clinical setting and then engages in the coaching process for the improvement of a specific skill, such as procedural training [41]. (b) Academic coaching: coaches guide learners to achieve their fullest potential by indirectly evaluating performance via review of objective assessments [41], such as: o self-reflection o specific, measurable, achievable, relevant and time-based (SMART) goal setting o the development of comprehensive study plans with deliberate use of effective learning strategies, including spaced retrieval practice and elaboration o self-care ▪ Teaching faculty members supported the streamlined and collaborative approach. Academic coaches offered timely oversight and early identification of students requiring support [42].
**Instruction**	▪ None of the articles defined instruction. ▪ According to the UNESCO International Bureau of Education, instruction is defined as “the creation and implementation of purposefully developed plans for guiding the process by which learners gain knowledge and understanding, and develop skills, attitudes, appreciations and values” [43].
**Supervision**	▪ May be viewed as “an intervention, a working alliance, a method, a process and a professional activity” [44]. ▪ May also be conceived of as “a joint endeavour in which a practitioner, with the help of a supervisor, attends to their clients, themselves as part of their client practitioner relationships and the wider systemic context, and by so doing, improves the quality of their work, transforms their client relationships, continuously develops themselves, their practice and the wider profession” [45]. ▪ Clinical supervision has been defined as the “provision of guidance and feedback on matters of personal, professional and educational development in the context of a trainee’s experience of providing safe and appropriate patient care” [46].
**Reflective Writing**	▪ Allows physicians the opportunity to reflect on their actions, recognise how their thoughts, feelings and emotions affect decision-making processes, clinical reasoning and professionalism that impact belief systems and shape PIF [47, 48].
**Group Reflections**	▪ Enhance holistic and collaborative learning [49, 50]. ▪ A means of determining the nature of the ‘takeaway’ from a specific learning interaction and boosting engagement. ▪ Access the hidden curriculum through facilitating discussions and self-reflection, providing insight into unspoken norms and values which influence clinical reasoning. ▪ Integrate diverse viewpoints into individual understandings of medical practice.
**Reflexivity**	▪ Described as a level of consciousness of ‘cultural, political, social, linguistic and ideologic’ origins of one’s own and others’ voice and perspective. ▪ Increases awareness of how personal values and beliefs interconnect with social and environmental contexts.

More recent notions of the mentoring umbrella suggest that it also contains assessment elements and support of self-care.

**Fig. 5:**
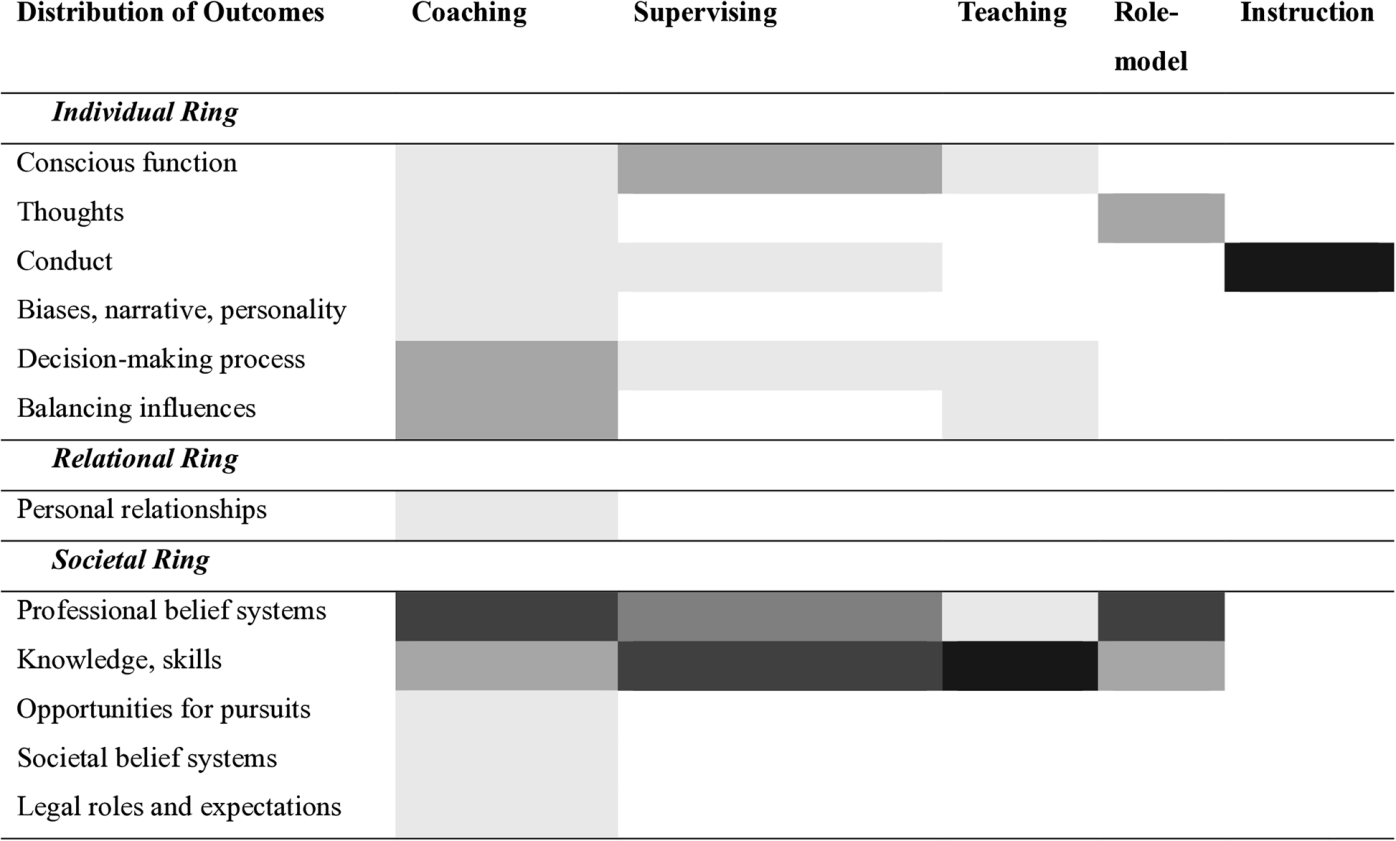
A heat map of the impact of key elements of the Mentoring Umbrella on the Individual, Relational and Societal rings

These constituent parts are prioritised according to their ability to meet the mentees needs, goals and individual and contextual considerations over the course of the mentoring process. Each element of the mentoring umbrella plays a particular role, not simply in the development of clinical knowledge and skills, but also in shaping their belief systems and moulding thinking. We use a heat map (Fig. 5) to illustrate the part played by the different aspects of the mentoring umbrella on the Individual, Relational and Societal Rings.

The darker colours correspond to greater frequency of use whilst lighter colours reflect a less significant role in the blend of support. For example, instruction dominated the blend of support from the mentoring umbrella in the moulding of conduct with smaller roles for coaching and supervision. Similarly, an equal mix of coaching and role modelling dominated the inculcation of professional beliefs with lesser effects on supervision and a very small role for teaching. We will show that this blend evolves over time and with mentee development.

### Domain 2. Personalisation

The heat map suggests that in each stage of development, there is a ‘standard’ mix to address each aspect of the mentee’s development. Each stage of the mentoring process, defined by particular goals and required competencies, begin with a ‘standard’ mix of constituent factors of the mentoring umbrella. This blend is personalised to meet the evolving needs of the mentee and their individualised and contextualised considerations [7, 10]. We detail this in Table 3 for ease of review.

**Table 3:** Individualised and contextual considerations influencing the blending of the Mentoring Umbrella

Individualised	Contextual
• Belief systems [51] • Discernment: Willingness, judgment and balancing [51, 52] • Narratives: Working styles, opportunities [53], attitudes, emotions [54], experience, skills and goals, as well as demographic [54, 55], socio-cultural [56–58], ideological, contextual, and psycho-emotional features • Coping strategies: Psycho-emotional well-being and the adoption of reflective practice [59–61], as well as personal coping strategies [62–68], including level of resilience [21, 69] and the ability to cope with emotionally-rich experiences [70], failures [71], moments of crisis [72], disorienting experiences [73] and transitions [71, 74–80] • Developing competencies: Skills, knowledge, levels of engagement, decision-making and practice • Maturing relationship: Nature, quality, setting and progress of patient interactions • Meaning-making and psycho-emotional state: Reflections, insights, adaptation, development [81, 82] and available support [83]	• The formal curriculum, or “the actual course of study, the planned content, teaching, evaluation methods, syllabi, and other materials used in any educational setting, formal policy statements, regulations, expectations, and competencies for every educational cohort conceivable” [51, 84] • Clear ‘membership’ criteria [85] • Competency-based mentoring stages [85] • Curricula: Hidden, formal and informal curriculum [76, 86–95], working hours [96], rules [97], disciplinary consequences [98], programmes [99, 100], attention to PIF [95, 101, 102], administrative support [103], faculty training and evaluation [103, 104], access to personalised support and communication networks [72, 90, 105–107] • Desired characteristics: Organisational, training, professional and speciality expectations on norms, skills, values, objectives, support and assessment systems [3, 108] • Host organisation-related facets: Practice differences across different training sites; evolving expectations and stages of training; differences in support and assessment systems; and the programme’s belief systems and shared identity [108] • Practice standards: The programme’s timelines, professional standards [109, 110], codes of conduct, expectations [111, 112], implicit norms [113], sociocultural norms and legal requirements [47, 114–117] • Practice culture that is shaped by the programme’s hidden curriculum [76, 86–95], prevailing discourses [72, 90, 105–107], daily activities [88, 118, 119] and rites of passage [10, 76, 93, 120–125] • PAL mentoring support: Access to timely, individualised, context-specific and appropriate role modelling [126]; clinically-relevant tutoring catering for individual consideration; supervised immersion into the clinical practice that accommodates the individual’s narratives, experiences, contextual considerations and goals; timely and comprehensive guided reflections; individualised, necessary [127], prompt and constructive feedback that impact meaning-making; context-specific advice; stage-specific assessment-led coaching; and longitudinal, personalised, appropriate, timely, holistic mentored support and ‘instructional scaffolding’ of a structured mentoring programme [3] • PAL support structures: Accessible communication; flexible and personalised support mechanisms [128–132]; longitudinal training support that caters to the physician’s personal needs [133, 134], abilities [135] and changing contextual considerations [130, 136–138]; quality of the apprenticeship relationship [130, 131, 136, 138–147]; and the learning environment [134, 136, 138, 147, 148] • Faculty selection, including the desired characteristics, [130, 134, 136, 138, 139, 143–145, 149–158], training and experience [128, 131, 132, 134, 138, 142–144, 147, 152, 159–161], commitment to training roles, openness to feedback [140, 147, 155, 162] and skills [95, 139, 148–150, 152, 163] • Peer support [3] • Intensity: Waxing and waning nature of the intensity of clinical practice [95, 137, 145, 150, 151] and the number of complex, morally and/or ethically challenging cases • Mentoring trajectory [2, 3] • Mentoring approach [19, 23, 164] • Mentor and peer-mentor training [13] • Stakeholder influences: Mentee, peer-mentor, mentor and host organisation influences [13] • Communication [165], feedback and remediation pathways • Longitudinal assessments

These considerations shift the standard mix of teaching, coaching, role modelling and supervision routinely employed to aid knowledge- and skills-building and the inculcation of belief systems [7, 10]. The mentee’s individualised and contextual considerations, shifting belief systems and developing mentoring relationships and competencies sees mentoring, counselling and guided reflective cycles taking more prominent roles as the mentoring journey progresses.

### Domain 3. Shepherding

Personalisation of mentoring support must be contained within programme expectations [83] and consistent with the programme belief systems and contextual and current sociocultural considerations, or ‘social validation’ [2, 3, 27]. In addition, there must be ‘congruence’ with the mentee’s individual and contextual considerations and current self-concepts of identity and personhood [2, 3]. Supporting these value-based considerations and balancing ‘social validation’ [2, 3, 27] and ‘congruence’ is the mentoring tube. The mentoring tube is described as a “combination of a mapped training programme, the mentoring umbrella, trained faculty, structured assessments, guided reflections and supervised experiential learning within the curated learning environment aspects [that] support a structured and personalised Socialisation Process” [83]. The mentoring tube ushers PAL mentoring support and integrates longitudinal feedback [69], communication, guidance and assessment network data to guide the blending of the PAL mentoring support as mentoring relationships progress runs along the mentoring trajectory. This process is guided by stage-specific mentoring assessments and feedback loops [21] that call attention to the mentor’s skills, competencies, mentoring characteristics, experiences and training [108]. It also highlights the host organisation’s role [21, 166] in supporting mentor training, assessments [33] and longitudinal support of mentors and the mentoring project [167, 168].

### Domain 4. Professional identity formation

Identified through SEBA’s reiterative process [83], PIF is shaped by PAL mentoring support [27, 51, 108, 169]. However, to consistently influence elements of the Individual, Relational and Societal Rings featured in Fig. 5, there are two essential components that were uncovered. The first is an adaptive mentoring tube that evolves with changing individual and contextual considerations (Table 3) and enhanced ‘sensitivity*’* [170]; greater confidence [171–176] and self-efficacy [173, 175, 177–180]; better ability in navigating personal and professional networks [6, 7, 20, 21, 173–175, 177, 179, 181–192]; and enhanced decision-making [173, 174, 176, 181, 184, 185, 188, 190, 191, 193–198]. This process is also impacted by a deepening belief system, maturing mentoring relationships, competencies and the development of a shared identity that shapes a mentee’s PIF [13].

The second component is a structured CoP-like mentoring programme, replete with consistent mentoring borders, mentoring tube and access to PAL mentoring support provided by trained and informed mentors. This CoP-like mentoring programme attenuates the influence of external factors and allows the development of the programme’s own mentoring environment that supports the developing PIF and mentoring relationships.

### Stage 6 of SEBA: synthesis of SSR in SEBA

Synthesis of this discussion is based on the four funnelled domains guided by the Best Evidence Medical Education (BEME) Collaboration guide and the STORIES (STructured apprOach to the Reporting In healthcare education of Evidence Synthesis) statement [199, 200].

## Discussion

This SSR in SEBA affirms that successful programmes rely on PAL mentoring support to personalise the socialisation process within a CoP-like mentoring programme. The role of PAL mentoring support is complex and requires some delineation.

Perhaps most significantly, PAL mentoring support personalises the relatively rigid structures established by the CoP-like mentoring programme by providing the appropriate mix of elements of the mentoring umbrella to develop PIF and ensure an individualised mentoring experience within the confines of programme codes of conduct, legal and ethical principles and current sociocultural norms (practice standards) scaffolding the socialisation process.

On the surface, this personalisation of the mentoring process is most evident at the start of each mentoring stage. Standard use of teaching, coaching, role modelling and supervision in early mentoring stages are individualised and injected with purposeful role modelling, guided immersion into the work environment, supervised reflections, mentored meaning-making and timely feedback. Blending this support is in part guided by the competency assessments that bookend the mentoring stages, appraisal of the evolving mentoring relationships that platform mentoring progress and policing of practice standards. Uniquely, these assessments are intimately entwined and a part of PAL mentoring support. This is surmised from PAL mentoring support’s inclusion of remedial action and guidance on self-care that is interweaved with reflective cycles and debriefs. The incorporation of assessment, remedial and support measures within the PAL mentoring support system moves beyond attending to ‘social validation’ [2, 3, 27], ‘congruence’ and individual and contextual considerations and focuses upon maturation of the mentoring relationships and competencies that bring about better teamworking [6, 7, 20, 21, 173–175, 177, 179, 181–192] and patient care [173, 174, 176, 181, 184, 185, 188, 190, 191, 193–198]. These notions are made possible through six considerations.

One, mentors and host organisation share a common mental model and appreciation of the mentoring goals, timelines and progress. They must act in sync to blend the various elements of the mentoring umbrella. This blending process is possible, given the inherent overlap in the roles and functions of each of these facets of the mentoring umbrella (Table 2).

Two, personalising the constituent factors found at the start of each mentoring stage demands effective and longitudinal assessments that extend beyond merely the competency-based assessments that bookend the mentoring stages.

Three, to achieve the requisite heat map for each mentee at each stage of the mentoring process, there needs to be careful coordination of informal and formal, formative and summative assessments (Fig. 5). This requires that the mentors be trained, equipped with effective assessment tools and provided with time and resources to blend and direct personalised mentoring support and evaluations that account for individual and contextual considerations.

Four, the interplay between Cruess-ian requirements and PAL mentoring support brings to the fore the critical role of the host organisation. It is the host organisation that must provide mentors with training and protected time to meet their various responsibilities, ensure an effective mentoring environment and establish clear expectations and practice standards that mould mentoring practice. The host organisation must also ensure that the programme is well resourced, structured and able to contend with evolving demands.

Five, aligned expectations, common belief systems and a shared identity that grow with the instillation of professional, ethical, legal, societal, moral and programmatic belief systems accelerate the development of PIF. Sharing common beliefs systems built within a trusting mentoring relationship and nurturing environment hastens the adoption of new belief systems and expediates PIF. In turn, these changes feed back to the blending of the PAL mentoring support that seeks to support these changes.

Six, it is here that the CoP-like programme with its clear boundaries helps to attenuate the external influences on the evolving mentoring relationship. This also allows more effective assessments of progress and change and facilitates the direction and blending of the PAL mentoring support.

These features come together to bring about better teamworking [6, 7, 20, 21, 173–175, 177, 179, 181–192] and patient care [173, 174, 176, 181, 184, 185, 188, 190, 191, 193–198]. This, in turn, brings to fore six factors that blending depends on:

- The mentor’s skills at assessing, blending and supporting the mentoring relationship, together with the mentee’s changing individualised and contextual considerations.
- The mentee’s engagement with evaluations and their willingness to seek support, accept feedback and be guided over the course of their mentoring journey.
- The quality and nature of mentoring relationships.
- The availability and accessibility of communication, support and assessment channels to actuate changes.
- The resources available and the role of the host organisation in investing, guiding and overseeing the mentoring process.
- The nature and structure of the mentoring programme and environment.

However, the exact formula for effective blending remains elusive and does underline the role of the host organisation, mentor and mentee.

### Mentor role and mentee engagement

**Fig. 6:**
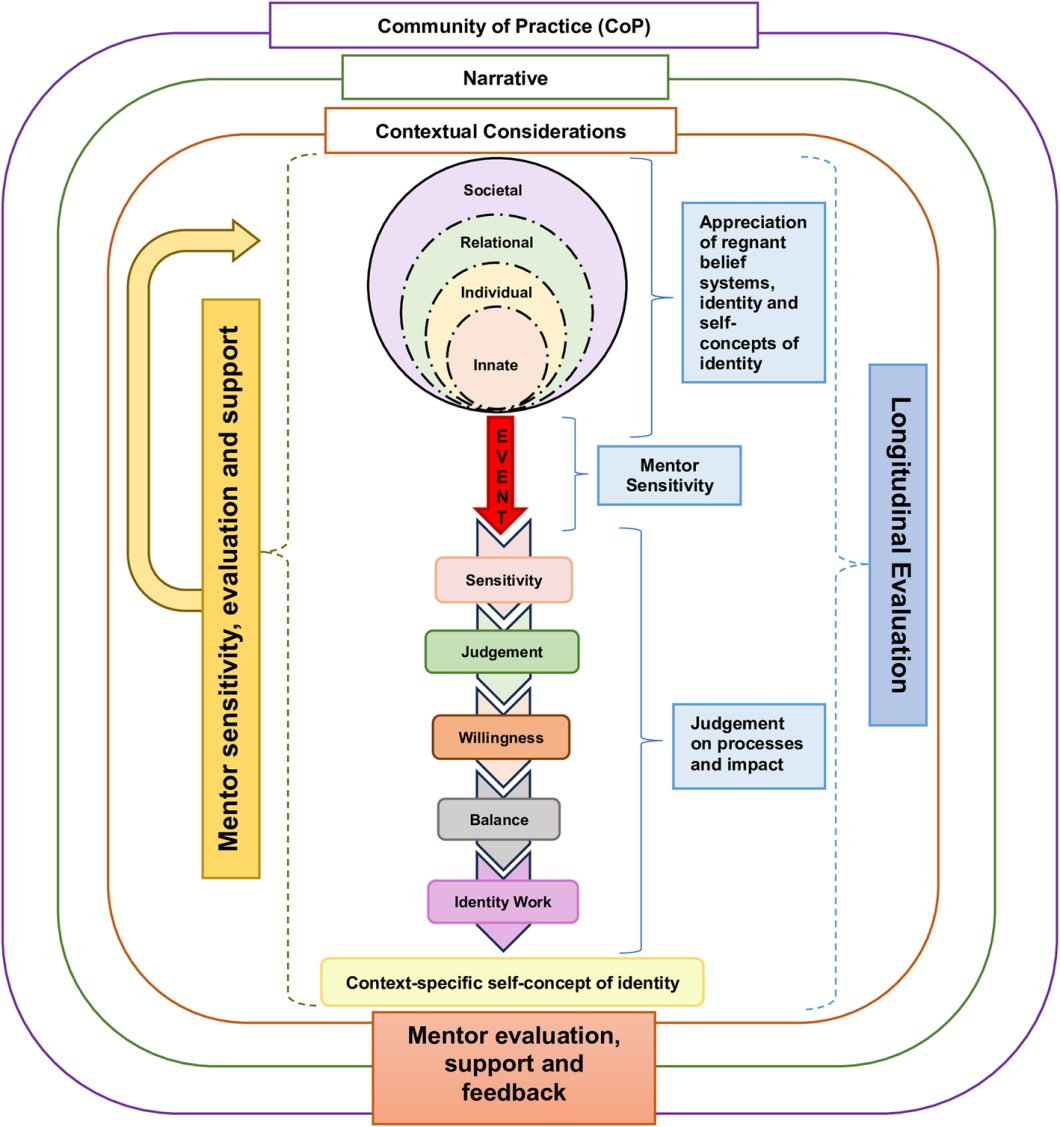
Adapted Krishna-Pisupati Model of the Mentor’s Role in the Blending of PAL Mentoring Support. This process is guided by the mentoring approach, belief systems, shared identity and the mentee’s current self-concepts of identity and personhood, narratives, individual and contextual considerations, congruence and social validations, mentoring resources, as well as the mentoring environment within the community of practice

Given that the success of the mentoring relationship depends on the mentor’s motivations, skills, experience, competency and availability to assess and diagnose the mentee’s needs and harness available support and communication structures to direct personalised support in a timely manner and review its effects, we adapt the KPM to scrutinise the mentor’s role (Fig. 6).

The role of the mentor is key and is situated in a good understanding of the mentee’s self-concept of personhood, identity, belief systems, goals and abilities, as well as an appreciation of current contextual, resource and programmatic considerations. To understand the mentee and their evolving needs and individualised and contextual considerations, the mentor must be able to nurture a trusting and open mentoring relationship where the mentee is able to share and discuss their concerns openly. To do so, much is reliant upon the mentor’s ability to detect ‘events’ or subsequent shifts in belief systems and blend mentoring support accordingly. ‘Mentor sensitivity’ precipitates mentor ‘evaluation, judgement and support’ of the mentee’s identity work. This support is shaped by the mentor’s ‘judgement’ of processes, the mentee’s individualised and contextual considerations and their impact, as well as the feedback received from the various stakeholders. The mentor’s experience, practical ability and clinical judgement, in addition to their training, assessment skills and ability to blend the mentoring umbrella, are critical to building the mentoring relationship and nurturing PIF.

These findings also draw attention to the importance of mentee engagement. From a practical perspective, mentors cannot provide continuous assessments of the mentee’s changing individualised and contextual considerations without active mentee engagement and reporting. The mentee must be able and willing to seek input from mentors and the host organisation when facing significant events or individualised and contextual considerations that may impact decision-making, conduct and practice. Mentees also play an important role in working with mentors and host organisations in nurturing trusting mentoring relationships and a safe mentoring environment, replete with robust communication, support and feedback pathways. Effective support is shaped when both parties are engaged and open to the involvement of the other.

### The host organisation and resources

The mentoring structure, culture and curation of the mentoring environment speak to the significant role played by the host organisation in ensuring that effective resources and support are available to mentors to meet their responsibilities. This includes ensuring robust mentoring boundaries, mentor training, protected time for mentoring and longitudinal support of mentors and mentees. The host organisation must ensure effective oversight of mentoring relationships, mentor development and the mentoring programme.

**Fig. 7:**
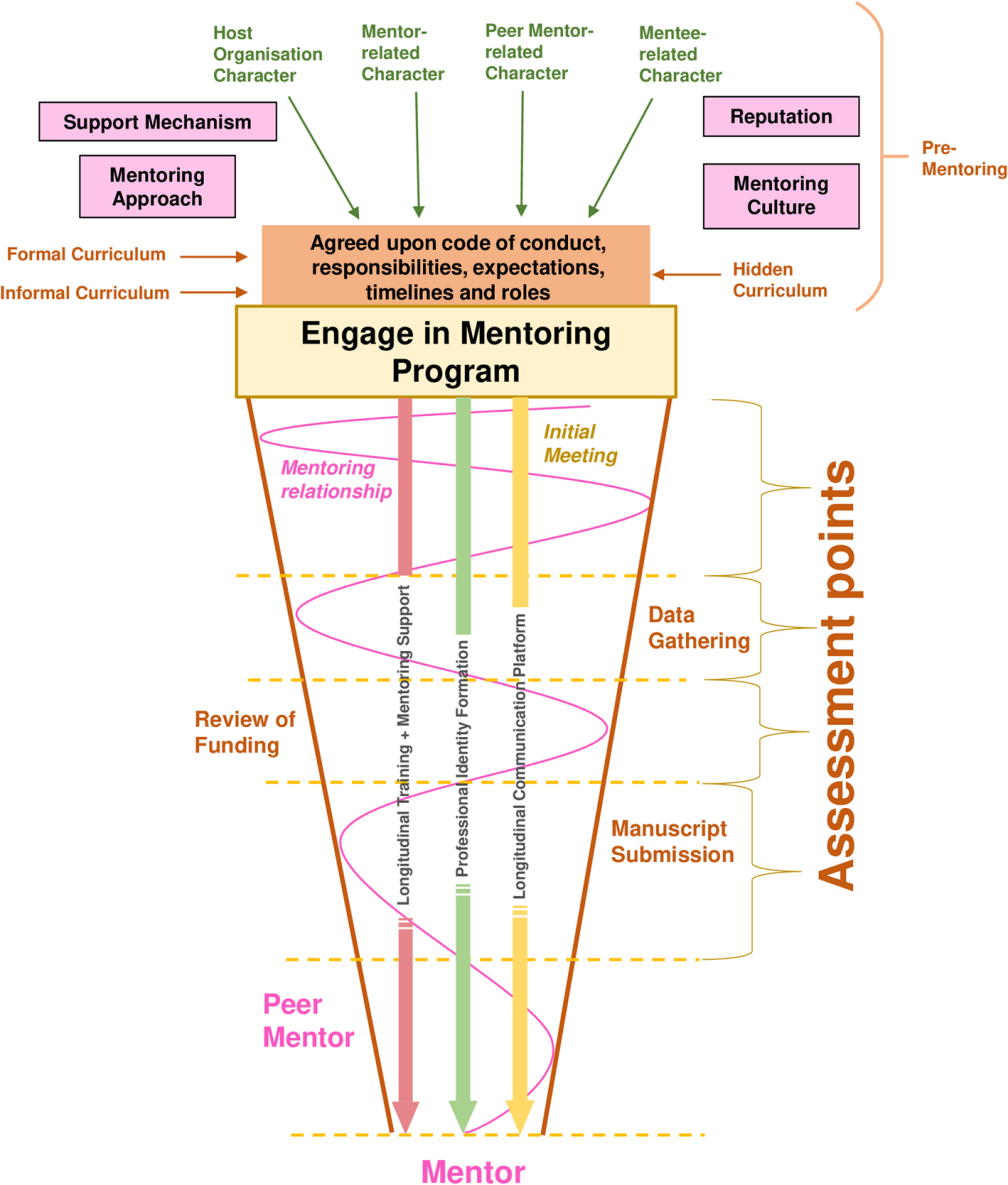
The Mentoring Structure, its Key Elements and its Intertwined Relationships

The success of the host organisation in supporting the mentoring relationship and mentee development relies on active engagement with the other stakeholders. The host organisation relies on the mentor to operationalise support and this relationship must be robust enough for the mentor to seek support from the host organisation. Data suggests that this support and effective blending of mentoring nurture a sense of belonging and affiliation with the profession, as well as buttress the acculturation and identification with “a particular society or group by internalising its values and norms” [12, 201]. These features and how they work together are summarised in Fig. 7.

- The **orange funnel boundaries** represent practice standards that confine mentoring support within programme expectations, professional standards and programme expectations.
- The **yellow vertical arrow** represents the longitudinal communication platform.
- The **red vertical arrow** represents the PAL mentoring support.
- The **green vertical arrow** represents the mentee’s PIF.
- The **pink spiral line** reveals the mentoring trajectory.
- The **assessment points** inform the programme’s support mechanisms.

However, how mentors assess and determine the blend of support to be provided and a clear appreciation of how elements of the mentoring umbrella influence the socialisation process remain unclear. Similarly, how PAL mentoring facilitates the development of mentoring relationships and the environment also requires further thought. In addition, the impact of mentoring support on the mentoring dynamics or the quality and nature of the mentoring relationship should be the focus of further studies.

### Limitations

This SSR combines data from medical students, residents and senior clinicians. These backgrounds differ significantly. The focus on largely North American and European practices, articles in English and defined date range collectively limit our findings’ generalisability. These factors underscore the need for context-specific interrogation of the terms used to define the different elements within the mentoring umbrella, in view of current practice variations. This may explain the lack of data on the effects of the mentoring umbrella on different elements of the Innate Ring. These gaps also reiterate the variable meaning of the terms and the manner that some of these elements are considered in different cultures and practices.

## Conclusion

The findings from our SEBA-guided SSR reveal that the success of mentoring in honing PIF is rooted in its ability to blend role modelling, supervision, mentoring, coaching and teaching with self-care, guided reflection, apprenticeship and assessment to meet the individual needs and changing circumstances of the mentee. The findings further emphasise the importance of:

- assessing and supporting the mentoring umbrella [166–168, 202].
- mentor and mentee engagement [25, 203].
- building trusting, enduring and personalised relationships [8, 15, 17, 108].
- addressing gaps in mentee and mentor training and support over the course of the mentoring process.
- the nature and level of personalised support [33, 69] which can take the form of portfolios [25, 203, 204], mentoring diaries and group [47, 48] and individual reflections [2, 169, 205] to supplement assessments, guided debriefs and reflective cycles.
- the impact of active support of mentoring relationships that ought to be mapped.
- ensuring the structuring of mentor training [22].
- nurturing a supportive mentoring environment [21].

These insights reflect the need to support the complex personal mentoring relationships and PIF. Whilst individual reviews of each of these elements have been ongoing, the focus of our coming work will focus on the immediate impact of mentoring support on the stakeholders as we continue to engage in this critical aspect of medical education.

## Supplementary Information

Additional File 1. Full Search Strategy

## Data Availability

The datasets supporting the conclusions of this article are included within the article and its additional files.

## Abbreviations

PIF: Professional Identity Formation
PAL Mentoring Support: Personalised, Appropriate and Longitudinal Mentoring Umbrella-based Support
SSR: Systematic Scoping Review
SEBA: Systematic Evidence-Based Approach
KPM: Krishna-Pisupati Model of Professional Identity Formation
RToP: Ring Theory of Personhood
CoP: Community of Practice
PICOS: Population, Intervention, Comparison, Outcome, Study Design
BEME: Best Evidence Medical Education
STORIES: STructured apprOach to the Reporting In healthcare education of Evidence Synthesis

## References

[1] Krishna LKR, Tan LHE, Ong YT, Tay KT, Hee JM, Chiam M, et al. Enhancing mentoring in palliative care: an evidence based mentoring framework. J Med Educ Curric Dev. 2020;7:2382120520957649. 33015366 10.1177/2382120520957649 PMC7517982

[2] Krishna LKR, Pisupati A, Ong YT, Teo KJH, Teo MYK, Venktaramana V, et al. Assessing the effects of a mentoring program on professional identity formation. BMC Med Educ. 2023;23(1):799. 37880728 10.1186/s12909-023-04748-6 PMC10601320

[3] Krishna LKR, Pisupati A, Teo KJH, Teo MYK, Quek CWN, Chua KZY, et al. Professional identity formation amongst peer-mentors in a research-based mentoring programme. BMC Med Educ. 2023;23(1):787. 37875886 10.1186/s12909-023-04718-y PMC10598986

[4] Lin J, Chew YR, Toh YP, Krishna LKR. Mentoring in nursing: an integrative review of commentaries, editorials, and perspectives papers. Nurse Educ. 2018;43(1):E1–5. 10.1097/NNE.0000000000000389 28492413

[5] Toh YP, Karthik R, Teo CC, Suppiah S, Cheung SL, Krishna LKR. Toward mentoring in palliative social work: a narrative review of mentoring programs in social work. Am J Hosp Palliat Care. 2018;35(3):523–31. 28641444 10.1177/1049909117715216

[6] Krishna LKR, Yaazhini, Tay KT, Tan B, Chong J, Ching A, et al. Educational roles as a continuum of mentoring’s role in medicine – a systematic review and thematic analysis of educational studies from 2000 to 2018. BMC Med Educ. 2019;19(1):439. 31775732 10.1186/s12909-019-1872-8 PMC6882248

[7] Krishna LKR, Tay KT, Yap HW, Koh ZYK, Ng YX, Ong YT, et al. Combined novice, near-peer, e-mentoring palliative medicine program: a mixed method study in Singapore. PLoS ONE. 2020;15(6):e0234322. 32502180 10.1371/journal.pone.0234322 PMC7274408

[8] Wahab M, Ikbal M, Jingting W, Wesley L, Kanesvaran R, Krishna LKR. Creating effective interprofessional mentoring relationships in palliative care- lessons from medicine, nursing, surgery and social work. J Palliat Care Med. 2016;6(6):290.

[9] Krishna LKR, Toh YP, Mason S, Kanesvaran R. Mentoring stages: a study of undergraduate mentoring in palliative medicine in Singapore. PLoS ONE. 2019;14(4):e0214643. 31017941 10.1371/journal.pone.0214643 PMC6481808

[10] Cruess RL, Cruess SR, Boudreau JD, Snell L, Steinert Y. A schematic representation of the professional identity formation and socialization of medical students and residents: a guide for medical educators. Acad Med. 2015;90(6):718–25. 25785682 10.1097/ACM.0000000000000700

[11] Barab SA. An introduction to the special issue: Designing for virtual communities in the service of learning. Inf Soc. 2003;19(3):197–201.

[12] Cruess R, Cruess S, Boudreau J, Snell L, Steinert Y. Reframing medical education to support professional identity formation. Acad Med. 2014;89(11):1446–51. 25054423 10.1097/ACM.0000000000000427

[13] Toh RQE, Koh KK, Lua JK, Wong RSM, Quah ELY, Panda A, et al. The role of mentoring, supervision, coaching, teaching and instruction on professional identity formation: a systematic scoping review. BMC Med Educ. 2022;22(1):531. 35804340 10.1186/s12909-022-03589-z PMC9270794

[14] Wesley L, Ikbal M, Jingting W, Wahab M, Yeam C. Towards a practice guided evidence based theory of mentoring in palliative care. J Palliat Care Med. 2017;7(1):296.

[15] Toh YP, Lam B, Soo J, Chua KLL, Krishna LKR. Developing palliative care physicians through mentoring relationships. Palliat Med Care. 2017;4(1):1–6.

[16] Jingting W, Wahab M, Ikbal M, Wesley L, Kanesvaran R, Krishna LKR. Toward an interprofessional mentoring program in palliative care - a review of undergraduate and postgraduate mentoring in medicine, nursing, surgery and social work. J Palliat Care Med. 2016;6(6):292.

[17] Sng JH, Pei Y, Toh YP, Peh TY, Neo SH, Krishna LKR. Mentoring relationships between senior physicians and junior doctors and/or medical students: a thematic review. Med Teach. 2017;39(8):866–75. 28562193 10.1080/0142159X.2017.1332360

[18] Ikbal M, Jingting W, Wahab M, Kanesvaran R, Krishna LKR. Mentoring in palliative medicine: guiding program design through thematic analysis of mentoring in internal medicine between 2000 and 2015. J Palliat Care Med. 2017;7(5):318.

[19] Tan YS, Teo SWA, Pei Y, Sng JH, Yap HW, Toh YP, et al. A framework for mentoring of medical students: thematic analysis of mentoring programmes between 2000 and 2015. Adv Health Sci Educ Theory Pract. 2018;23(4):671–97. 29550907 10.1007/s10459-018-9821-6

[20] Qiao Ting Low C, Toh YL, Teo SWA, Toh YP, Krishna LKR. A narrative review of mentoring programmes in general practice. Educ Prim Care. 2018;29(5):259–67. 30059278 10.1080/14739879.2018.1474723

[21] Hee JM, Yap HW, Ong ZX, Quek SQM, Toh YP, Mason S, et al. Understanding the mentoring environment through thematic analysis of the learning environment in medical education: a systematic review. J Gen Intern Med. 2019;34(10):2190–9. 31011975 10.1007/s11606-019-05000-y PMC6816739

[22] Sheri K, Too JYJ, Chuah SEL, Toh YP, Mason S, Krishna LKR. A scoping review of mentor training programs in medicine between 1990 and 2017. Med Educ Online. 2019;24(1):1555435. 31671284 10.1080/10872981.2018.1555435 PMC6327936

[23] Tan B, Toh YL, Toh YP, Kanesvaran R, Krishna LKR. Extending mentoring in palliative medicine-systematic review on peer, near-peer and group mentoring in general medicine. J Palliat Care Med. 2017;7(6):323.

[24] Lim YX, Quah ELY, Chua KZY, Lin CKR, Govindasamy R, Ong SM, et al. A systematic scoping review on dignity assessment tools. J Pain Symptom Manage. 2024;67(4):e263–84. 38092260 10.1016/j.jpainsymman.2023.12.008

[25] Lim AJS, Hong DZ, Pisupati A, Ong YT, Yeo JYH, Chong EJX, et al. Portfolio use in postgraduate medical education: a systematic scoping review. Postgrad Med J. 2023;99(1174):913–27. 36961214 10.1093/postmj/qgac007

[26] Koh EYH, Koh KK, Renganathan Y, Krishna LKR. Role modelling in professional identity formation: a systematic scoping review. BMC Med Educ. 2023;23(1):194. 36991373 10.1186/s12909-023-04144-0 PMC10052869

[27] Ong YT, Quek CWN, Pisupati A, Loh EKY, Venktaramana V, Chiam M, et al. Mentoring future mentors in undergraduate medical education. PLoS ONE. 2022;17(9):e0273358. 36108091 10.1371/journal.pone.0273358 PMC9477267

[28] Krishna LKR, Alsuwaigh R. Understanding the fluid nature of personhood - the ring theory of personhood. Bioethics. 2015;29(3):171–81. 24547934 10.1111/bioe.12085

[29] Peters MDJ, Godfrey CM, Khalil H, McInerney P, Parker D, Soares CB. Guidance for conducting systematic scoping reviews. Int J Evid Based Healthc. 2015;13(3):141–6. 26134548 10.1097/XEB.0000000000000050

[30] Wong G, Greenhalgh T, Westhorp G, Buckingham J, Pawson R. RAMESES publication standards: Meta-narrative reviews. BMC Med. 2013;11(1):20. 23360661 10.1186/1741-7015-11-20 PMC3558334

[31] Virginia B, Victoria C. Using thematic analysis in psychology. Qual Res Psychol. 2006;3(2):77–101.

[32] Boyatzis RE. Transforming qualitative information: thematic analysis and code development. Thousand Oaks, California: SAGE; 1998. p. 184.

[33] Ng YX, Koh ZYK, Yap HW, Tay KT, Tan XH, Ong YT, et al. Assessing mentoring: a scoping review of mentoring assessment tools in internal medicine between 1990 and 2019. PLoS ONE. 2020;15(5):e0232511. 32384090 10.1371/journal.pone.0232511 PMC7209188

[34] Noblit GW, Hare RD. Meta-ethnography: synthesizing qualitative studies. California: Sage; 1988. p. 88.

[35] France EF, Uny I, Ring N, Turley RL, Maxwell M, Duncan EAS, et al. A methodological systematic review of meta-ethnography conduct to articulate the complex analytical phases. BMC Med Res Methodol. 2019;19(1):35. 30777031 10.1186/s12874-019-0670-7 PMC6380066

[36] Agzarian J, Blackmon SH, Cassivi SD, Shen KR, Shargall Y. Moving to the other side of the table-transitioning from residency to faculty and the value of mentorship. J Thorac Dis. 2019;11(Suppl 7):S1018–21. 31183185 10.21037/jtd.2019.04.03 PMC6535474

[37] Spencer J. Learning and teaching in the clinical environment. BMJ. 2003;326(7389):591–4. 12637408 10.1136/bmj.326.7389.591 PMC1125480

[38] Smith JM. Surgeon coaching: why and how. J Pediatr Orthop. 2020;40:S33–7. 32502069 10.1097/BPO.0000000000001541

[39] Mikhaiel JP, Pollack J, Buck E, Williams M, Lott A, Penner JC, et al. Graduating with honors in resilience: creating a whole new doctor. Glob Adv Health Med. 2020;9:2164956120976356. 33329942 10.1177/2164956120976356 PMC7720294

[40] Stalmeijer RE, Dolmans DHJM, Wolfhagen IHAP, Scherpbier AJJA. Cognitive apprenticeship in clinical practice: can it stimulate learning in the opinion of students? Adv Health Sci Educ Theory Pract. 2009;14(4):535–46. 18798005 10.1007/s10459-008-9136-0 PMC2744784

[41] Margaret Wolff, Morgan H, Jackson J, Skye E, Hammoud M, Ross PT. Academic coaching: insights from the medical student’s perspective. Med Teach. 2020;42(2):172–7. 31630595 10.1080/0142159X.2019.1670341

[42] Lee ICJ, Koh H, Lai SH, Hwang NC. Academic coaching of medical students during the COVID-19 pandemic. Med Educ. 2020;54(12):1184–5. 32531804 10.1111/medu.14272

[43] Glossory of curriculum terminology. 2013. https://unesdoc.unesco.org/ark:/48223/pf0000223059.locale=en. Cited 6 June 2024.

[44] Barham D, de Beer W, Clark H. The role of professional supervision for palliative care doctors in New Zealand: a quantitative survey of attitudes and experiences. NZ Med J. 2019;132(1501):10–20. 31465323

[45] Hawkins P, McMahon A. Supervision in the helping professions. 5th ed. London: McGraw-Hill Education; 2020. p. 354.

[46] Sue Kilminster DCJG, Brian J. AMEE guide 27: effective educational and clinical supervision. Med Teach. 2007;29(1):2–19. 17538823 10.1080/01421590701210907

[47] Phua GLG, Owyong JLJ, Leong ITY, Goh S, Somasundaram N, Poon EYL, et al. A systematic scoping review of group reflection in medical education. BMC Med Educ. 2024;24(1):398. 38600515 10.1186/s12909-024-05203-w PMC11007913

[48] Lim JY, Ong SYK, Ng CYH, Chan KLE, Wu SYEA, So WZ, et al. A systematic scoping review of reflective writing in medical education. BMC Med Educ. 2023;23(1):12. 36624494 10.1186/s12909-022-03924-4 PMC9830881

[49] Farrington R, Collins L, Fisher P, Danquah A, Sergeant J. Clinical debrief: learning and well-being together. Clin Teach. 2019;16(4):329–34. 31309726 10.1111/tct.13055 PMC6900240

[50] Barbour RS. Making sense of focus groups. Med Educ. 2005;39(7):742–50. 15960795 10.1111/j.1365-2929.2005.02200.x

[51] Teo MYK, Ibrahim H, Lin CKR, Hamid NABA, Govindasamy R, Somasundaram N, et al. Mentoring as a complex adaptive system – a systematic scoping review of prevailing mentoring theories in medical education. BMC Med Educ. 2024;24(1):726. 38970020 10.1186/s12909-024-05707-5 PMC11225364

[52] Sarraf-Yazdi SPA, Goh CK, Ong YT, Toh YR, Goh SPL, Krishna LKR. A scoping review and theory-informed conceptual model of professional identity formation in medical education. Med Educ Online. 2024;58(10):1151–65. 10.1111/medu.15399 38597258

[53] Crampton PES, Afzali Y. Professional identity formation, intersectionality and equity in medical education. Med Educ. 2021;55(2):140–2. 33179338 10.1111/medu.14415

[54] Wyatt TR, Balmer D, Rockich-Winston N, Chow CJ, Richards J, Zaidi Z. Whispers and shadows’: a critical review of the professional identity literature with respect to minority physicians. Med Educ. 2021;55(2):148–58. 33448459 10.1111/medu.14295

[55] Chow CJ, Byington CL, Olson LM, Ramirez KPG, Zeng S, López AM. A conceptual model for understanding academic physicians’ performances of identity: findings from the University of Utah. Acad Med. 2018;93(10):1539–49. 29794525 10.1097/ACM.0000000000002298 PMC6156991

[56] Ong EK, Krishna LKR. Perspective from Singapore. Asian Bioeth Rev. 2014;6(4):420–7.

[57] Ong EK, Krishna LKR, Neo PSH. The sociocultural and ethical issues behind the decision for artificial hydration in a young palliative patient with recurrent intestinal obstruction. Ethics Med. 2015;31(1):39–51.

[58] Surbone A, Baider L. Personal values and cultural diversity. J Med Person. 2013;11(1):11–8.

[59] Dréano-Hartz S, Rhondali W, Ledoux M, Ruer M, Berthiller J, Schott A-M, et al. Burnout among physicians in palliative care: impact of clinical settings. Palliat Support Care. 2016;14(4):402–10. 26462566 10.1017/S1478951515000991

[60] Koh MYH, Hum AYM, Khoo HS, Ho AHY, Chong PH, Ong WY, et al. Burnout and resilience after a decade in palliative care: what survivors have to teach us. A qualitative study of palliative care clinicians with more than 10 years of experience. J Pain Symptom Manage. 2020;59(1):105–15. 31465787 10.1016/j.jpainsymman.2019.08.008

[61] Lehto RH, Heeter C, Forman J, Shanafelt T, Kamal A, Miller P, et al. Hospice employees’ perceptions of their work environment: a focus group perspective. Int J Environ Res Public Health. 2020;17(17):6147. 32847036 10.3390/ijerph17176147 PMC7503310

[62] Koh MYH, Chong PH, Neo PSH, Ong YJ, Yong WC, Ong WY, et al. Burnout, psychological morbidity and use of coping mechanisms among palliative care practitioners: a multi-centre cross-sectional study. Palliat Med. 2015;29(7):633–42. 25829444 10.1177/0269216315575850

[63] Back AL, Steinhauser KE, Kamal AH, Jackson VA. Building resilience for palliative care clinicians: an approach to burnout prevention based on individual skills and workplace factors. J Pain Symptom Manage. 2016;52(2):284–91. 26921494 10.1016/j.jpainsymman.2016.02.002

[64] Kavalieratos D, Siconolfi DE, Steinhauser KE, Bull J, Arnold RM, Swetz KM, et al. It is like heart failure. It is chronic… and it will kill you: a qualitative analysis of burnout among hospice and palliative care clinicians. J Pain Symptom Manage. 2017;53(5):901–e910901. 28063867 10.1016/j.jpainsymman.2016.12.337 PMC5410187

[65] Ercolani G, Varani S, Peghetti B, Franchini L, Malerba MB, Messana R, et al. Burnout in home palliative care: what is the role of coping strategies? J Palliat Care. 2020;35(1):46–52. 30727827 10.1177/0825859719827591

[66] Tan BYQ, Kanneganti A, Lim LJH, Tan M, Chua YX, Tan L, et al. Burnout and associated factors among health care workers in Singapore during the COVID-19 pandemic. J Am Med Dir Assoc. 2020;21(12):1751–e17581755. 33256955 10.1016/j.jamda.2020.09.035 PMC7534835

[67] Dijxhoorn A-FQ, Brom L, van der Linden YM, Leget C, Raijmakers NJ. Prevalence of burnout in healthcare professionals providing palliative care and the effect of interventions to reduce symptoms: a systematic literature review. Palliat Med. 2021;35(1):6–26. 33063609 10.1177/0269216320956825

[68] Teo YH, Peh TY, Abdurrahman A, Lee A, Chiam M, Fong W, et al. A modified delphi approach to enhance nurturing of professionalism in postgraduate medical education in Singapore. Singap Med J. 2021;65(6):313–25. 10.11622/smedj.2021224 PMC11232710 34823327

[69] Lim SYS, Koh EYH, Tan BJX, Toh YP, Mason S, Krishna LKR. Enhancing geriatric oncology training through a combination of novice mentoring and peer and near-peer mentoring: a thematic analysis ofmentoring in medicine between 2000 and 2017. J Geriatr Oncol. 2020;11(4):566–75. 31699675 10.1016/j.jgo.2019.09.008

[70] Kilbertus F, Ajjawi R, Archibald DB. You’re not trying to save somebody from death: learning as becoming in palliative care. Acad Med. 2018;93(6):929–36. 29116978 10.1097/ACM.0000000000001994

[71] Burford B. Group processes in medical education: learning from social identity theory. Med Educ. 2012;46(2):143–52. 22239328 10.1111/j.1365-2923.2011.04099.x

[72] Jarvis-Selinger S, Pratt DD, Regehr G. Competency is not enough: integrating identity formation into the medical education discourse. Acad Med. 2012;87(9):1185–90. 22836834 10.1097/ACM.0b013e3182604968

[73] Sawatsky AP, Nordhues HC, Merry SP, Bashir MU, Hafferty FW. Transformative learning and professional identity formation during international health electives: a qualitative study using grounded theory. Acad Med. 2018;93(9):1381–90. 29596082 10.1097/ACM.0000000000002230

[74] Soo J, Brett-MacLean P, Cave MT, Oswald A. At the precipice: a prospective exploration of medical students’ expectations of the pre-clerkship to clerkship transition. Adv Health Sci Educ Theory Pract. 2016;21(1):141–62. 26164285 10.1007/s10459-015-9620-2

[75] Stuart E, O’Leary D, Rowntree R, Carey C, O’Rourke L, O’Brien E, et al. Challenges in experiential learning during transition to clinical practice: a comparative analysis of reflective writing assignments during general practice, paediatrics and psychiatry clerkships. Med Teach. 2020;42(11):1275–82. 32776857 10.1080/0142159X.2020.1803250

[76] Kay D, Berry A, Coles NA. What experiences in medical school trigger professional identity development? Teach Learn Med. 2019;31(1):17–25. 29608109 10.1080/10401334.2018.1444487

[77] Hamstra SJ, Woodrow SI, Mangrulkar RS. Feeling pressure to stay late: Socialisation and professional identity formation in graduate medical education. Med Educ. 2008;42(1):7–9. 18181842 10.1111/j.1365-2923.2007.02958.x

[78] Wilson I, Cowin LS, Johnson M, Young H. Professional identity in medical students: pedagogical challenges to medical education. Teach Learn Med. 2013;25(4):369–73. 24112208 10.1080/10401334.2013.827968

[79] Wald HS, White J, Reis SP, Esquibel AY, Anthony D. Grappling with complexity: medical students’ reflective writings about challenging patient encounters as a window into professional identity formation. Med Teach. 2019;41(2):152–60. 29944035 10.1080/0142159X.2018.1475727

[80] Matthews R, Smith-Han K, Nicholson H. From physiotherapy to the army: negotiating previously developed professional identities in mature medical students. Adv Health Sci Educ Theory Pract. 2020;25(3):607–27. 31701305 10.1007/s10459-019-09942-0

[81] Coetzee SK, Klopper HC. Compassion fatigue within nursing practice: a concept analysis. Nurs Health Sci. 2010;12(2):235–43. 20602697 10.1111/j.1442-2018.2010.00526.x

[82] McCann IL, Pearlman LA. Constructivist self-development theory: a theoretical framework for assessing and treating traumatized college students. J Am Coll Health. 1992;40(4):189–96. 1583241 10.1080/07448481.1992.9936281

[83] Teo KJH, Teo MYK, Pisupati A, Ong RSR, Goh CK, Seah CHX, et al. Assessing professional identity formation (PIF) amongst medical students in oncology and palliative medicine postings: a SEBA guided scoping review. BMC Palliat Care. 2022;21(1):200. 36397067 10.1186/s12904-022-01090-4 PMC9673314

[84] Wear D, Skillicorn J. Hidden in plain sight: the formal, informal, and hidden curricula of a psychiatry clerkship. Acad Med. 2009;84(4):451–8. 19318777 10.1097/ACM.0b013e31819a80b7

[85] Krishna L, Toh YPMS, Kanesvaran R. Mentoring stages: a study of undergraduate mentoring in palliative medicine in Singapore. PLoS ONE. 2019;14(4):e0214643. 31017941 10.1371/journal.pone.0214643 PMC6481808

[86] Gilligan C, Loda T, Junne F, Zipfel S, Kelly B, Horton G, et al. Medical identity; perspectives of students from two countries. BMC Med Educ. 2020;20(1):420. 33172441 10.1186/s12909-020-02351-7 PMC7654572

[87] Wang XM, Swinton M, You JJ. Medical students’ experiences with goals of care discussions and their impact on professional identity formation. Med Educ. 2019;53(12):1230–42. 31750573 10.1111/medu.14006

[88] Witman Y. What do we transfer in case discussions? The hidden curriculum in medicine…. Perspect Med Educ. 2014;3(2):113–23. 24366760 10.1007/s40037-013-0101-0 PMC3976482

[89] Hafferty FW, Franks R. The hidden curriculum, ethics teaching, and the structure of medical education. Acad Med. 1994;69(11):861–71. 7945681 10.1097/00001888-199411000-00001

[90] Whitehead C, Kuper A, Freeman R, Grundland B, Webster F. Compassionate care? A critical discourse analysis of accreditation standards. Med Educ. 2014;48(6):632–43. 24807439 10.1111/medu.12429

[91] Seoane L, Tompkins LM, De Conciliis A, Boysen PG. 2nd. Virtues education in medical school: the foundation for professional formation. Ochsner J. 2016;16(1):50–5. 27046405 PMC4795502

[92] Gaufberg E, Bor D, Dinardo P, Krupat E, Pine E, Ogur B, et al. In pursuit of educational integrity: Professional identity formation in the Harvard Medical School Cambridge Integrated Clerkship. Perspect Biol Med. 2017;60(2):258–74. 29176087 10.1353/pbm.2017.0032

[93] Monrouxe LV. Identity, identification and medical education: why should we care? Med Educ. 2010;44(1):40–9. 20078755 10.1111/j.1365-2923.2009.03440.x

[94] Chuang AW, Nuthalapaty FS, Casey PM, Kaczmarczyk JM, Cullimore AJ, Dalrymple JL, et al. To the point: reviews in medical education - taking control of the hidden curriculum. Am J Obstet Gynecol. 2010;203(4):316.E311–316.E316. 10.1016/j.ajog.2010.04.035 20541735

[95] Kenny NP, Mann KV, MacLeod H. Role modeling in physicians’ professional formation: reconsidering an essential but untapped educational strategy. Acad Med. 2003;78(12):1203–10. 14660418 10.1097/00001888-200312000-00002

[96] Cope A, Bezemer J, Mavroveli S, Kneebone R. What attitudes and values are incorporated into self as part of professional identity construction when becoming a surgeon? Acad Med. 2017;92(4):544–9. 28351068 10.1097/ACM.0000000000001454

[97] Au A. Online physicians, offline patients. Int J Sociol Soc Policy. 2018;38(5–6):474–83.

[98] Al-Abdulrazzaq D, Al-Fadhli A, Arshad A. Advanced medical students’ experiences and views on professionalism at Kuwait University. BMC Med Educ. 2014;14(1):150. 25056201 10.1186/1472-6920-14-150 PMC4118198

[99] Byszewski A, Hendelman W, McGuinty C, Moineau G. Wanted: role models - medical students’ perceptions of professionalism. BMC Med Educ. 2012;12(1):115. 23153359 10.1186/1472-6920-12-115 PMC3537482

[100] Kavas MV, Demirören M, Koşan AMA, Karahan ST, Yalim NY. Turkish students’ perceptions of professionalism at the beginning and at the end of medical education: a cross-sectional qualitative study. Med Educ Online. 2015;20(1):26614. 25795382 10.3402/meo.v20.26614 PMC4368711

[101] Smith SE, Tallentire VR, Cameron HS, Wood SM. The effects of contributing to patient care on medical students’ workplace learning. Med Educ. 2013;47(12):1184–96. 24206152 10.1111/medu.12217

[102] Rosenblum ND, Kluijtmans M, Cate OT. Professional identity formation and the clinician–scientist: a paradigm for a clinical career combining two distinct disciplines. Acad Med. 2016;91(12):1612–7. 27254011 10.1097/ACM.0000000000001252

[103] Meyer EM, Zapatka S, Brienza RS. The development of professional identity and the formation of teams in the Veterans affairs Connecticut healthcare system’s center of excellence in primary care education program (CoEPCE). Acad Med. 2015;90(6):802–9. 25551857 10.1097/ACM.0000000000000594

[104] Birden H, Glass N, Wilson I, Harrison M, Usherwood T, Nass D. Teaching professionalism in medical education: a best evidence medical education (BEME) systematic review. BEME Guide 25 Med Teach. 2013;35(7):e1252–1266. 23829342 10.3109/0142159X.2013.789132

[105] Frost HD, Regehr G. I am a doctor: negotiating the discourses of standardization and diversity in professional identity construction. Acad Med. 2013;88(10):1570–7. 23969361 10.1097/ACM.0b013e3182a34b05

[106] Rodríguez C, López-Roig S, Pawlikowska T, Schweyer F-X, Bélanger E, Pastor-Mira MA, et al. The influence of academic discourses on medical students’ identification with the discipline of family medicine. Acad Med. 2015;90(5):660–70. 25406604 10.1097/ACM.0000000000000572

[107] MacLeod A. Caring, competence and professional identities in medical education. Adv Health Sci Educ Theory Pract. 2011;16(3):375–94. 21188513 10.1007/s10459-010-9269-9

[108] Venktaramana V, Ong YT, Yeo JW, Pisupati A, Krishna LKR. Understanding mentoring relationships between mentees, peer and senior mentors. BMC Med Educ. 2023;23(1):76. 36717909 10.1186/s12909-023-04021-w PMC9887801

[109] Fischer MA, Haley H-L, Saarinen CL, Chretien KC. Comparison of blogged and written reflections in two medicine clerkships. Med Educ. 2011;45(2):166–75. 21208262 10.1111/j.1365-2923.2010.03814.x

[110] Kern DE, Wright SM, Carrese JA, Lipkin M Jr., Simmons JM, Novack DH, et al. Personal growth in medical faculty: a qualitative study. West J Med. 2001;175(2):92–8. 11483549 10.1136/ewjm.175.2.92 PMC1071495

[111] Kimmons R, Veletsianos G. The fragmented educator 2.0: Social networking sites, acceptable identity fragments, and the identity constellation. Comput Educ. 2014;72:292–301.

[112] Gosselink MJ. Medical weblogs: advocacy for positive cyber role models. Clin Teach. 2011;8(4):245–8. 22085000 10.1111/j.1743-498X.2011.00483.x

[113] Fieseler C, Meckel M, Ranzini G. Professional personae - how organizational identification shapes online identity in the workplace. J Comput Mediat Commun. 2014;20(2):153–70.

[114] Maghrabi RO, Oakley RL, Nemati HR. The impact of self-selected identity on productive or perverse social capital in social network sites. Comput Hum Behav. 2014;33:367–71.

[115] Hojat M, Vergare MJ, Maxwell K, Brainard G, Herrine SK, Isenberg GA, et al. The devil is in the third year: a longitudinal study of erosion of empathy in medical school. Acad Med. 2009;84(9):1182–91. 19707055 10.1097/ACM.0b013e3181b17e55

[116] Newton BW, Barber L, Clardy J, Cleveland E, O’Sullivan P. Is there hardening of the heart during medical school? Acad Med. 2008;83(3):244–9. 18316868 10.1097/ACM.0b013e3181637837

[117] Kaczmarczyk JM, Chuang A, Dugoff L, Abbott JF, Cullimore AJ, Dalrymple J, et al. E-professionalism: a new frontier in medical education. Teach Learn Med. 2013;25(2):165–70. 23530680 10.1080/10401334.2013.770741

[118] Warmington S, McColl G. Medical student stories of participation in patient care-related activities: the construction of relational identity. Adv Health Sci Educ Theory Pract. 2017;22(1):147–63. 27235124 10.1007/s10459-016-9689-2

[119] Foster K, Roberts C. The heroic and the villainous: a qualitative study characterising the role models that shaped senior doctors’ professional identity. BMC Med Educ. 2016;16(1):206. 27530252 10.1186/s12909-016-0731-0 PMC4986406

[120] Hendelman W, Byszewski A. Formation of medical student professional identity: categorizing lapses of professionalism, and the learning environment. BMC Med Educ. 2014;14(1):139. 25004924 10.1186/1472-6920-14-139 PMC4102062

[121] Sternszus R, Boudreau JD, Cruess RL, Cruess SR, Macdonald ME, Steinert Y. Clinical teachers’ perceptions of their role in professional identity formation. Acad Med. 2020;95(10):1594–9. 32271232 10.1097/ACM.0000000000003369

[122] Jarvis-Selinger S, MacNeil KA, Costello GRL, Lee K, Holmes CL. Understanding professional identity formation in early clerkship: a novel framework. Acad Med. 2019;94(10):1574–80. 31192797 10.1097/ACM.0000000000002835

[123] Sadeghi Avval Shahr H, Yazdani S, Afshar L. Professional socialization: an analytical definition. J Med Ethics Hist Med. 2019;12:17. 32328230 10.18502/jmehm.v12i17.2016 PMC7166248

[124] Brody H, Doukas D, Professionalism. A framework to guide medical education. Med Educ. 2014;48(10):980–7. 25200018 10.1111/medu.12520

[125] Irby DM, Hamstra SJ. Parting the clouds: three professionalism frameworks in medical education. Acad Med. 2016;91(12):1606–11. 27119331 10.1097/ACM.0000000000001190

[126] Liang JZND, Vijayprasanth Raveendran V, Teo MYK, Quah ELY, Chua KZY, Lua JK, Owyong JLJ, Vijayan AV, Hamid NABA, Yeoh TT, Ong EK, Phua GLG, Mason S, Fong W, Lim C, Woong N, Ong SYK, Krishna LKR. The impact of online education during the covid-19 pandemic on the professional identity formation of medical students: a systematic scoping review. PLoS ONE. 2024;19(1):e0296367. 38181035 10.1371/journal.pone.0296367 PMC10769105

[127] Quek CWN, Ong RRS, Wong RSM, Chan SWK, Chok AK, Shen GS, et al. Systematic scoping review on moral distress among physicians. BMJ Open. 2022;12(9):e064029. 10.1136/bmjopen-2022-064029 PMC9442489 36691160

[128] Klamen DL, Williams R, Hingle S. Getting real: aligning the learning needs of clerkship students with the current clinical environment. Acad Med. 2019;94(1):53–8. 30157091 10.1097/ACM.0000000000002434

[129] Morris MC, Hennessy M, Conlon KC, Ridgway PF. Evaluation of a subintern role: action over observation for final-year medical students in surgery. J Surg Educ. 2015;72(5):862–7. 25921190 10.1016/j.jsurg.2015.03.003

[130] Régo P, Peterson R, Callaway L, Ward M, O’Brien C, Donald K. Using a structured clinical coaching program to improve clinical skills training and assessment, as well as teachers’ and students’ satisfaction. Med Teach. 2009;31(12):e586–595. 19995160 10.3109/01421590903193588

[131] Remmen R, Denekens J, Scherpbier A, Hermann I, van der Vleuten C, Royen PV, et al. An evaluation study of the didactic quality of clerkships. Med Educ. 2000;34(6):460–4. 10792687 10.1046/j.1365-2923.2000.00570.x

[132] Wallenburg I, Hopmans CJ, Buljac-Samardzic M, Den Hoed PT, Ijzermans JNM. Repairing reforms and transforming professional practices: a mixed-methods analysis of surgical training reform. J Prof Organ. 2016;3(1):86–102.

[133] Gheasuddin AN, Misra R, Patel J. Use of an apprenticeship model to facilitate prescribing learning on clinical placements. Med Teach. 2022;44(8):940. 34587855 10.1080/0142159X.2021.1984412

[134] Wearne SM, Butler L, Jones JA. Educating registrars in your practice. Aust Fam Physician. 2016;45(5):274–7. 27166460

[135] Lim-Dunham JE, Ensminger DC, McNulty JA, Hoyt AE, Chandrasekhar AJ. A vertically integrated online radiology curriculum developed as a cognitive apprenticeship: impact on student performance and learning. Acad Radiol. 2016;23(2):252–61. 26719161 10.1016/j.acra.2015.09.018

[136] Bettin KA. The role of mentoring in the professional identity formation of medical students. Orthop Clin North Am. 2020;52(1):61–8. 33222985 10.1016/j.ocl.2020.08.007

[137] Kittmer T, Hoogenes J, Pemberton J, Cameron BH. Exploring the hidden curriculum: a qualitative analysis of clerks’ reflections on professionalism in surgical clerkship. Am J Surg. 2013;205(4):426–33. 23313441 10.1016/j.amjsurg.2012.12.001

[138] Thomas C, Plumblee L, Dieffenbaugher S, Talley C. Teaching on rounds and in small groups. Surg Clin North Am. 2021;101(4):555–63. 34242599 10.1016/j.suc.2021.05.003

[139] Baerheim A, Thesen J. Medical students’ evaluation of preceptorship in general practice in Vestlandet. Tidsskr nor Laegeforen. 2003;123(16):2271–3. 14508552

[140] Brown J, Reid H, Dornan T, Nestel D. Becoming a clinician: Trainee identity formation within the general practice supervisory relationship. Med Educ. 2020;54(11):993–1005. 32350873 10.1111/medu.14203

[141] Cloyd J, Holtzman D, O’Sullivan P, Sammann A, Tendick F, Ascher N. Operating room assist: Surgical mentorship and operating room experience for preclerkship medical students. J Surg Educ. 2008;65(4):275–82. 18707660 10.1016/j.jsurg.2008.04.002

[142] Golden BP, Henschen BL, Gard LA, Ryan ER, Evans DB, Bierman J, et al. Learning to be a doctor: medical students’ perception of their roles in longitudinal outpatient clerkships. Patient Educ Couns. 2018;101(11):2018–24. 30122264 10.1016/j.pec.2018.08.003

[143] Harris GD, Professionalism. Part ii - teaching and assessing the learner’s professionalism. Fam Med. 2004;36(6):390–2. 15181546

[144] Leeuw J-VD, Buwalda HGAR, Wieringa-De Waard N, Van Dijk M. Learning from a role model: a cascade or whirlpool effect? Med Teach. 2015;37(5):482–9. 25213300 10.3109/0142159X.2014.956061

[145] Peer KS. Professional identity formation: considerations for athletic training education. Athl Train Educ J. 2016;11(3):125–6.

[146] Sheu L, Goglin S, Collins S, Cornett P, Clemons S, O’Sullivan PS. How do clinical electives during the clerkship year influence career exploration? A qualitative study. Teach Learn Med. 2021;34(2):187–97. 33792448 10.1080/10401334.2021.1891545

[147] Huda N, Faden L, Wilson CA, Plouffe RA, Li E, Saini MK, et al. The ebb and flow of identity formation and competence development in sub-specialty residents: study of a continuity training setting. 2020. 10.21203/rs.3.rs-24203/v1.

[148] Ratanawongsa N, Teherani A, Hauer KE. Third-year medical students’ experiences with dying patients during the internal medicine clerkship: a qualitative study of the informal curriculum. Acad Med. 2005;80(7):641–7. 15980080 10.1097/00001888-200507000-00006

[149] Abbey L, Willett R, Selby-Penczak R, McKnight R. Social learning: medical student perceptions of geriatric house calls. Gerontol Geriatr Educ. 2010;31(2):149–62. 20509061 10.1080/02701961003795771

[150] Alford CL, Currie DM. Introducing first-year medical students to clinical practice by having them shadow third-year clerks. Teach Learn Med. 2004;16(3):260–3. 15388382 10.1207/s15328015tlm1603_7

[151] Boudreau J, Macdonald ME, Steinert Y. Affirming professional identities through an apprenticeship: insights from a four-year longitudinal case study. Acad Med. 2014;89(7):1038–45. 24826856 10.1097/ACM.0000000000000293

[152] Côté L, Laughrea PA. Preceptors’ understanding and use of role modeling to develop the CanMEDS competencies in residents. Acad Med. 2014;89(6):934–9. 24871246 10.1097/ACM.0000000000000246

[153] Goldstein EA, MacLaren CF, Smith S, Mengert TJ, Maestas RR, Foy HM, et al. Promoting fundamental clinical skills: a competency-based college approach at the University of Washington. Acad Med. 2005;80(5):423–33. 15851451 10.1097/00001888-200505000-00003

[154] Hay A, Smithson S, Mann K, Dornan T. Medical students’ reactions to an experience-based learning model of clinical education. Perspect Med Educ. 2013;2(2):58–71. 23670698 10.1007/s40037-013-0061-4 PMC3656171

[155] Jones K, Reis S. Learning through vulnerability: a mentor-mentee experience. Ann Fam Med. 2010;8(6):552–5. 21060127 10.1370/afm.1165 PMC2975692

[156] Kalén S, Stenfors-Hayes T, Hylin U, Larm MF, Hindbeck H, Ponzer S. Mentoring medical students during clinical courses: a way to enhance professional development. Med Teach. 2010;32(8):e315–321. 20662566 10.3109/01421591003695295

[157] Nirodi P, El-Sayeh H, Henfrey H. Applying the apprenticeship model to psychiatry: an evaluation. Prog Neurol Psychiatry. 2018;22(1):25–9.

[158] Tariq M, Iqbal S, Haider SI, Abbas A. Using the cognitive apprenticeship model to identify learning strategies that learners view as effective in ward rounds. Postgrad Med J. 2021;97(1143):5–9. 32817495 10.1136/postgradmedj-2020-137519

[159] Behling F, Nasi-Kordhishti I, Haas P, Sandritter J, Tatagiba M, Herlan S. One-on-one mentoring for final year medical students during the neurosurgery rotation. BMC Med Educ. 2021;21(1):229. 33882933 10.1186/s12909-021-02657-0 PMC8061075

[160] Braniff C, Spence RA, Stevenson M, Boohan M, Watson P. Assistantship improves medical students’ perception of their preparedness for starting work. Med Teach. 2016;38(1):51–8. 26037743 10.3109/0142159X.2015.1045843

[161] Iwata K, Gill D. Learning through work: clinical shadowing of junior doctors by first year medical students. Med Teach. 2013;35(8):633–8. 23782048 10.3109/0142159X.2013.801552

[162] Balmer DF, Serwint JR, Ruzek SB, Giardino AP. Understanding paediatric resident-continuity preceptor relationships through the lens of apprenticeship learning. Med Educ. 2008;42(9):923–9. 18715490 10.1111/j.1365-2923.2008.03121.x

[163] Bleakley A. Pre-registration house officers and ward-based learning: a ‘new apprenticeship’ model. Med Educ. 2002;36(1):9–15. 11849519 10.1046/j.1365-2923.2002.01128.x

[164] Low CTY, Teo A, Toh YP, Krishna LKR. A narrative review of mentoring programs in general practice. Educ Prim Care. 2018;29(5):259–67. 30059278 10.1080/14739879.2018.1474723

[165] Venktaramana V, Loh EKY, Wong CJW, Yeo JW, Teo AYT, Chiam CSY, et al. A systematic scoping review of communication skills training in medical schools between 2000 and 2020. Med Teach. 2022;44(9):997–1006. 35653622 10.1080/0142159X.2022.2054693

[166] Chia EWY, Tay KT, Xiao S, Teo YH, Ong YT, Chiam M, et al. The pivotal role of host organizations in enhancing mentoring in internal medicine: a scoping review. J Med Educ Curric Dev. 2020;7:2382120520956647. 33062895 10.1177/2382120520956647 PMC7536487

[167] Cheong CWS, Chia EWY, Tay KT, Chua WJ, Lee FQH, Koh EYH, et al. A systematic scoping review of ethical issues in mentoring in internal medicine, family medicine and academic medicine. Adv Health Sci Educ Theory Pract. 2020;25(2):415–39. 31705429 10.1007/s10459-019-09934-0

[168] Kow CS, Teo YH, Teo YN, Chua KZY, Quah ELY, Kamal NHBA, et al. A systematic scoping review of ethical issues in mentoring in medical schools. BMC Med Educ. 2020;20(1):246. 32736552 10.1186/s12909-020-02169-3 PMC7395401

[169] Goh S, Wong RSM, Quah ELY, Chua KZY, Lim WQ, Ng ADR, et al. Mentoring in palliative medicine in the time of covid-19: a systematic scoping review. BMC Med Educ. 2022;22(1):359. 35545787 10.1186/s12909-022-03409-4 PMC9094135

[170] Carey EC, Weissman DE. Understanding and finding mentorship: a review for junior faculty. J Palliat Med. 2010;13(11):1373–9. 21091022 10.1089/jpm.2010.0091 PMC3000901

[171] Efstathiou JA, Drumm MR, Paly JP, Lawton DM, O’Neill RM, Niemierko A, et al. Long-term impact of a faculty mentoring program in academic medicine. PLoS ONE. 2018;13(11):e0207634. 30496199 10.1371/journal.pone.0207634 PMC6264475

[172] Phillips G, Lee D, Shailin S, O’Reilly G, Cameron P. The Pacific emergency medicine mentoring program: a model for medical mentoring in the Pacific region. Emerg Med Australas. 2019;31(6):1092–100. 31379098 10.1111/1742-6723.13366

[173] Reitz R, Mitchell S, Keel S. Attachment-informed mentorship. Fam Syst Health. 2017;35(4):498–504. 29283616 10.1037/fsh0000300

[174] Feeley AA, Feeley IH, Sheehan E, Carroll C, Queally J. Impact of mentoring for underrepresented groups in undergraduate medical education: a systematic review. J Surg Educ. 2024;81(3):353–66. 38160117 10.1016/j.jsurg.2023.11.015

[175] Hill SEM, Ward WL, Seay A, Buzenski J. The nature and evolution of the mentoring relationship in academic health centers. J Clin Psychol Med Settings. 2022;29(3):557–69. 35761033 10.1007/s10880-022-09893-6 PMC9243938

[176] Sharshadeep M, Venkatappa K. Qualitative study on perception of first-year medical undergraduates toward mentorship program. Natl J Physiol Pharm Pharmacol. 2019;9(9):884–92.

[177] Chen MM, Sandborg CI, Hudgins L, Sanford R, Bachrach LK. A multifaceted mentoring program for junior faculty in academic pediatrics. Teach Learn Med. 2016;28(3):320–8. 27054562 10.1080/10401334.2016.1153476 PMC5003054

[178] Liu C, Wang L, Qi R, Wang W, Jia S, Shang D, et al. Prevalence and associated factors of depression and anxiety among doctoral students: the mediating effect of mentoring relationships on the association between research self-efficacy and depression/anxiety. Psychol Res Behav Manag. 2019;12:195–208. 30962730 10.2147/PRBM.S195131 PMC6432885

[179] Meeuwissen SNE, Stalmeijer RE, Govaerts M. Multiple-role mentoring: mentors’ conceptualisations, enactments and role conflicts. Med Educ. 2019;53(6):605–15. 30723949 10.1111/medu.13811 PMC6590242

[180] Sood V, Wiggins W, Rodriguez A, Sigl D. Attitudes of newly hired medicine faculty regarding mentorship and developmental networks. Chron Mentor Coach. 2022;6(Spec Iss 15):624–9. 36713783 PMC9880633

[181] Badawy SM, Black V, Meier ER, Myers KC, Pinkney K, Hastings C, et al. Early career mentoring through the American society of pediatric hematology/oncology: lessons learned from a pilot program. Pediatr Blood Cancer. 2017;64(3):e26252. 10.1002/pbc.26252 PMC5685518 27616578

[182] Mohtady HA, Könings KD, Al-Eraky MM, Muijtjens AMM, Van Merriënboer JJG. High enthusiasm about long lasting mentoring relationships and older mentors. BMC Med Educ. 2019;19(1):364. 31547807 10.1186/s12909-019-1791-8 PMC6757421

[183] Li C, Veinot P, Mylopoulos M, Leung FH, Law M. The new mentee: exploring Gen Z women medical students’ mentorship needs and experiences. Clin Teach. 2023;21(3):e13697. 38050710 10.1111/tct.13697

[184] Nemeth A, Chisty A, Spagnoletti CL, Stankiewicz CA, Burant C, Ramani S. Exploring mentoring experiences, perceptions, and needs of general internal medicine clinician educators navigating academia: a mixed-methods study. J Gen Intern Med. 2021;36(5):1229–36. 33140271 10.1007/s11606-020-06310-2 PMC8131409

[185] Abudayyeh I, Tandon A, Wittekind SG, Rzeszut AK, Sivaram CA, Freeman AM, et al. Landscape of mentorship and its effects on success in cardiology. JACC Basic Transl Sci. 2020;5(12):1181–6. 33426375 10.1016/j.jacbts.2020.09.014 PMC7775959

[186] Cellini MM, Serwint JR, D’Alessandro DM, Schulte EE, Osman C. Evaluation of a speed mentoring program: achievement of short-term mentee goals and potential for longer-term relationships. Acad Pediatr. 2017;17(5):537–43. 28040574 10.1016/j.acap.2016.12.012

[187] Margolis RD, Berenstain LK, Janosy N, Yanofsky S, Tackett S, Schwartz JM, et al. Grow and advance through intentional networking: a pilot program to foster connections within the women’s empowerment and leadership initiative in the society for pediatric anesthesia. Paediatr Anaesth. 2021;31(9):944–52. 34166544 10.1111/pan.14247

[188] Ogdie A, Sparks JA, Angeles-Han ST, Bush K, Castelino FV, Golding A, et al. Barriers and facilitators of mentoring for trainees and early career investigators in rheumatology research: current state, identification of needs, and road map to an inter-institutional adult rheumatology mentoring program. Arthritis Care Res (Hoboken). 2018;70(3):445–53. 28544766 10.1002/acr.23286 PMC5700864

[189] Pickering A, Patiño A, Garbern SC, Abu-Jubara D, Digenakis A, Rodigin A, et al. Building a virtual community of practice for medical students: the global emergency medicine student leadership program. JACEP Open. 2021;2(6):e12591. 35005703 10.1002/emp2.12591 PMC8716569

[190] Raharjo SB, Mustika R, Lydia A, Yanni M, Sulastomo H, Zhuhra RT, et al. Trainees’ perceptions and expectations of formal academic mentoring during the COVID-19 pandemic in Indonesian cardiology residency programs. J Educ Eval Health Prof. 2021;18:19. 34399567 10.3352/jeehp.2021.18.19 PMC8616722

[191] Sawatsky AP, Parekh N, Muula AS, Mbata I, Bui T. Cultural implications of mentoring in sub-saharan Africa: a qualitative study. Med Educ. 2016;50(6):657–69. 27170084 10.1111/medu.12999

[192] Zhou SY, Balakrishna A, Nyhof-Young J, Javeed I, Robinson LA. What do participants value in a diversity mentorship program? Perspectives from a Canadian medical school. Equal Divers Incl. 2021;40(8):947–59.

[193] Pololi LH, Evans AT, Civian JT, Gibbs BK, Gillum LH, Brennan RT. A novel measure of good mentoring: testing its reliability and validity in four academic health centers. J Contin Educ Health Prof. 2016;36(4):263–8. 28350307 10.1097/CEH.0000000000000114

[194] Winfrey SR, Parameswaran P, Gerull KM, LaPorte D, Cipriano CA. Effective mentorship of women and underrepresented minorities in orthopaedic surgery: a mixed-methods investigation. JB JS Open Access. 2022;7(4):e2200053. 10.2106/JBJS.OA.22.00053 PMC9699573 36447495

[195] Ahmadmehrabi S, Farlow JL, Wamkpah NS, Esianor BI, Brenner MJ, Valdez TA, et al. New age mentoring and disruptive innovation - navigating the uncharted with vision, purpose, and equity. AMA Otolaryngol Head Neck Surg. 2021;147(4):389–94. 10.1001/jamaoto.2020.5448 33538788

[196] Atlas AM, Seltzer ES, Watters A, Riley B, Chan T. A global perspective of mentorship in medical schools: systematic review from 2014 to 2019. Med Sci Educ. 2021;31(2):969–77. 34457937 10.1007/s40670-021-01252-8 PMC8368923

[197] Henry-Noel N, Bishop M, Gwede CK, Petkova E, Szumacher E. Mentorship in medicine and other health professions. J Cancer Educ. 2019;34(4):629–37. 29691796 10.1007/s13187-018-1360-6

[198] Zuzuarregui JR, Wu C, Hohler AD. Promoting careers in neurology: mentorship of medical students. Semin Neurol. 2018;38(4):413–7. 30125895 10.1055/s-0038-1666988

[199] Haig A, Dozier M. BEME guide 3: systematic searching for evidence in medical education—part 2: constructing searches. Med Teach. 2003;25(5):463–84. 14522667 10.1080/01421590310001608667

[200] Frei E, Stamm M, Buddeberg-Fischer B. Mentoring programs for medical students - a review of the PubMed literature 2000–2008. BMC Med Educ. 2010;10(1):32. 20433727 10.1186/1472-6920-10-32 PMC2881011

[201] Iserson K. Talking about professionalism through the lens of professional identity. AEM Educ Train. 2018;3(1):105–12. 30680357 10.1002/aet2.10307 PMC6339534

[202] Chua WJ, Cheong CWS, Lee FQH, Koh EYH, Toh YP, Mason S, et al. Structuring mentoring in medicine and surgery. A systematic scoping review of mentoring programs between 2000 and 2019. J Contin Educ Health Prof. 2020;40(3):158–68. 32898120 10.1097/CEH.0000000000000308

[203] Ting JJQ, Phua GLG, Hong DZ, Lam BKY, Lim AJS, Chong EJX, et al. Evidence-guided approach to portfolio-guided teaching and assessing communications, ethics and professionalism for medical students and physicians: a systematic scoping review. BMJ Open. 2023;13(3):e067048. 10.1136/bmjopen-2022-067048 PMC10069516 36977542

[204] Hong DZ, Lim AJS, Tan R, Ong YT, Pisupati A, Chong EJX, et al. A systematic scoping review on portfolios of medical educators. J Med Educ Curric Dev. 2021;8:23821205211000356. 35187262 10.1177/23821205211000356 PMC8855455

[205] Tay KT, Tan XH, Tan LHE, Vythilingam D, Chin AMC, Loh V, et al. A systematic scoping review and thematic analysis of interprofessional mentoring in medicine from 2000 to 2019. J Interprof Care. 2021;35(6):927–39. 33290115 10.1080/13561820.2020.1818700

