# Supplementary figures and images for: A systematic scoping review of mentoring support on professional identity formation
Source: BMC Med Educ. 2024 Nov 27;24:1380. doi: 10.1186/s12909-024-06357-3 (PMC11600620; doi:10.1186/s12909-024-06357-3)

**Additional File 1. Full Search Strategy**

| **PubMed** | 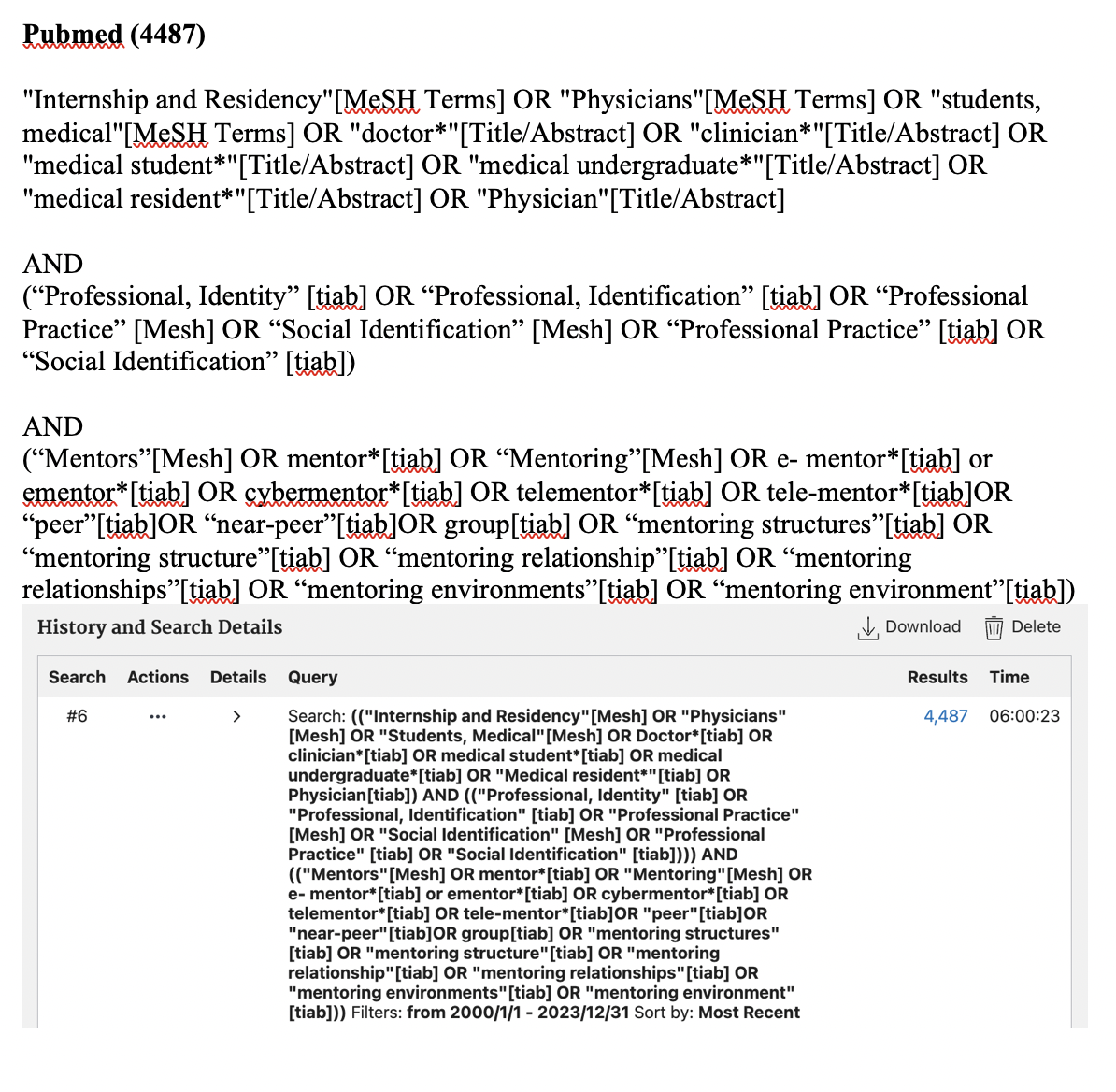 |
| --- | --- |
| **Embase** | 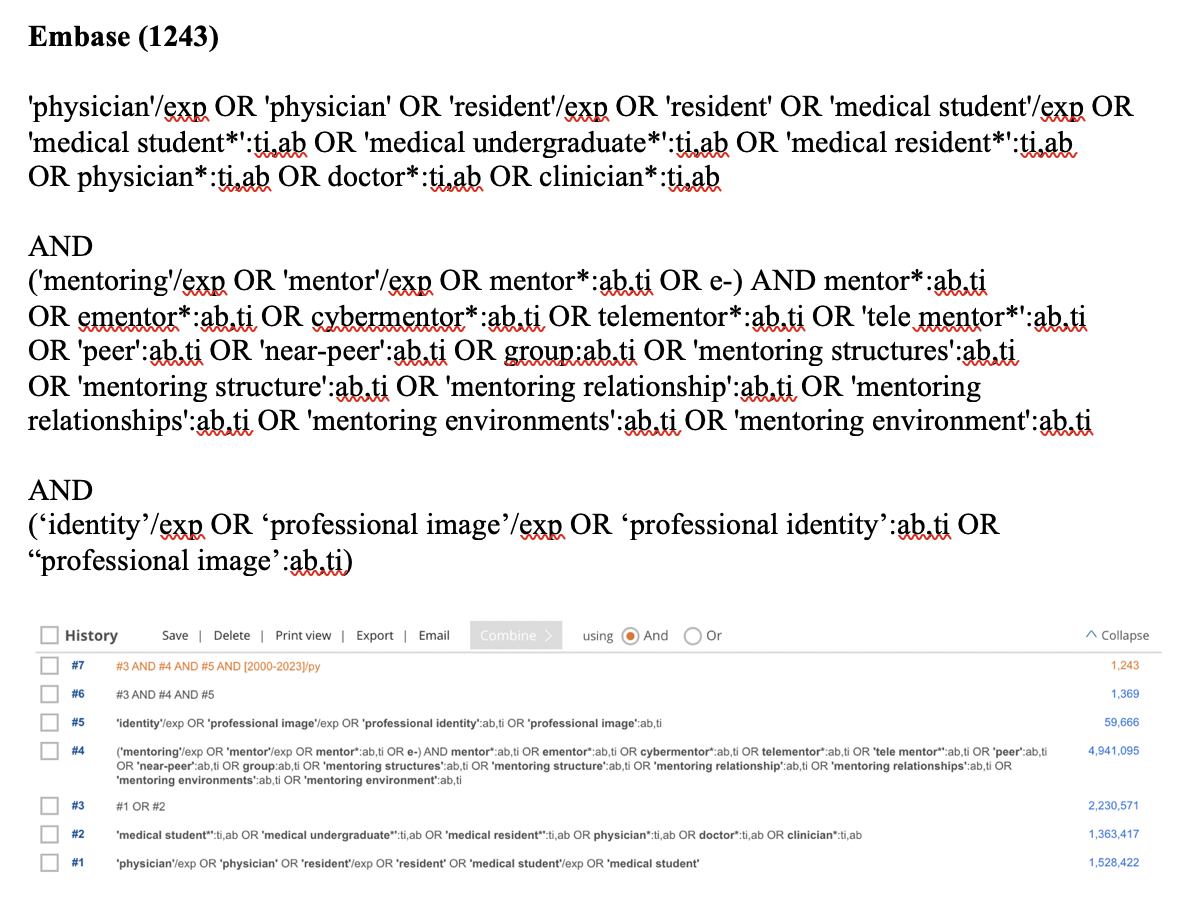 |
| **Scopus** | 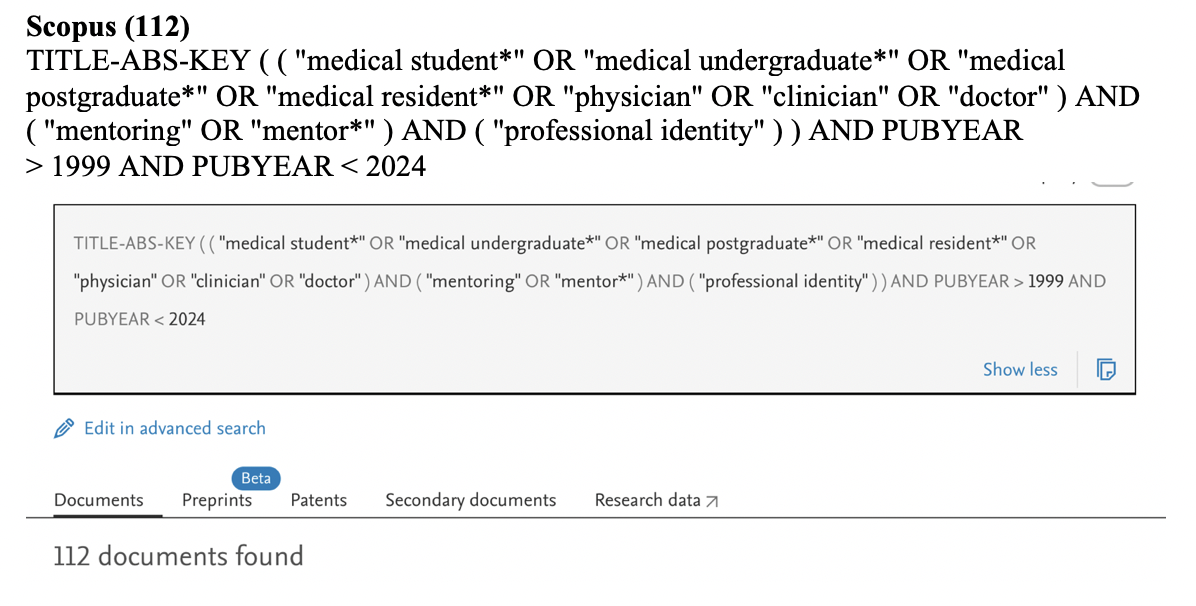 |
| **ERIC** | 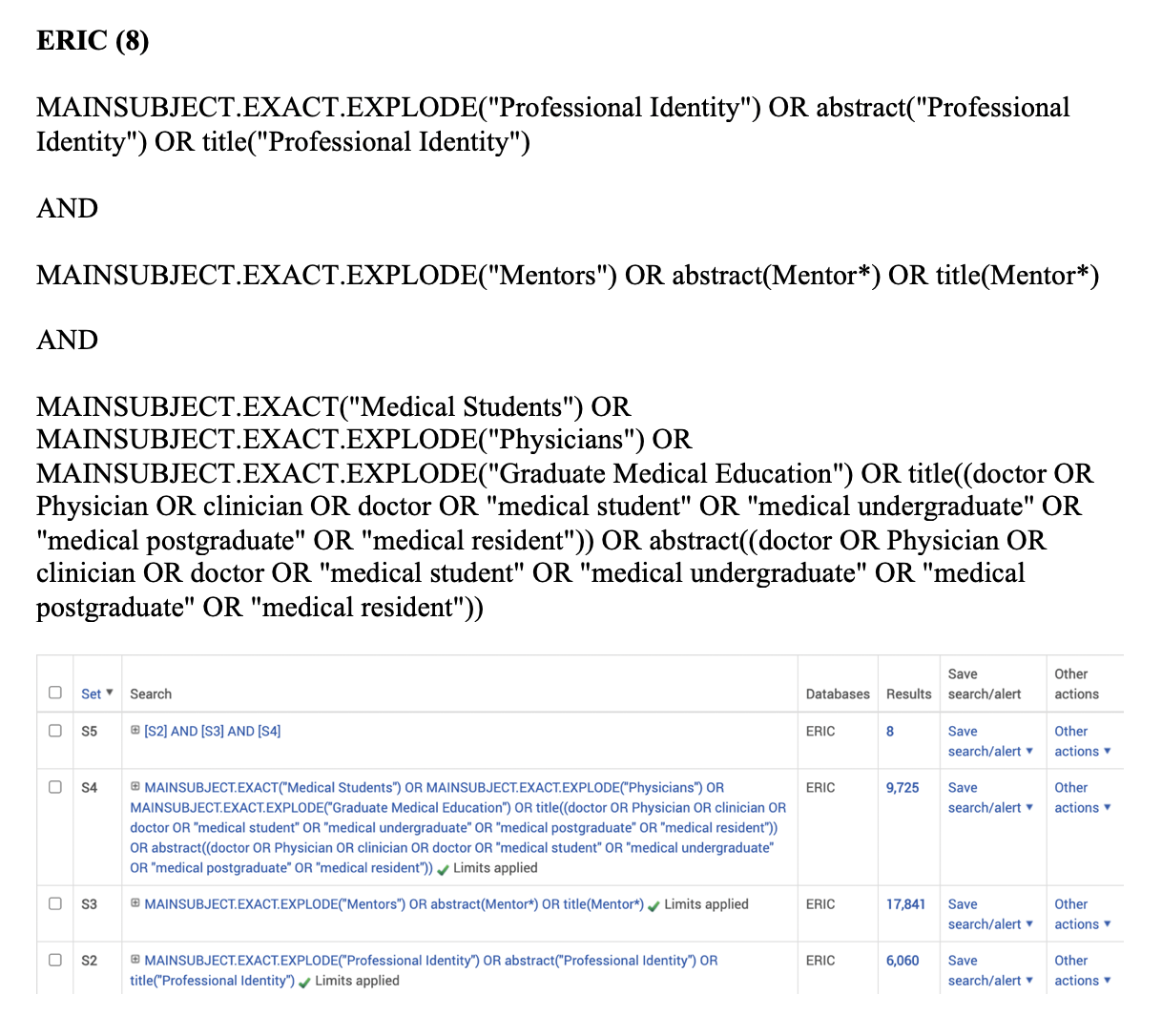 |

Supplement: Supplementary file 1 — Additional File 1. Full Search Strategy [file 12909_2024_6357_MOESM1_ESM.docx]
